# Naturally Engineered Maturation of Cardiomyocytes

**DOI:** 10.3389/fcell.2017.00050

**Published:** 2017-05-05

**Authors:** Gaetano J. Scuderi, Jonathan Butcher

**Affiliations:** Meinig School of Biomedical Engineering, Cornell UniversityIthaca, NY, USA

**Keywords:** natural engineering, cardiomyocyte maturation, mechanical stimulation, electrical stimulation, non-cardiomyocyte signaling, extracellular matrix signaling, engineered heart tissue

## Abstract

Ischemic heart disease remains one of the most prominent causes of mortalities worldwide with heart transplantation being the gold-standard treatment option. However, due to the major limitations associated with heart transplants, such as an inadequate supply and heart rejection, there remains a significant clinical need for a viable cardiac regenerative therapy to restore native myocardial function. Over the course of the previous several decades, researchers have made prominent advances in the field of cardiac regeneration with the creation of *in vitro* human pluripotent stem cell-derived cardiomyocyte tissue engineered constructs. However, these engineered constructs exhibit a functionally immature, disorganized, fetal-like phenotype that is not equivalent physiologically to native adult cardiac tissue. Due to this major limitation, many recent studies have investigated approaches to improve pluripotent stem cell-derived cardiomyocyte maturation to close this large functionality gap between engineered and native cardiac tissue. This review integrates the natural developmental mechanisms of cardiomyocyte structural and functional maturation. The variety of ways researchers have attempted to improve cardiomyocyte maturation *in vitro* by mimicking natural development, known as natural engineering, is readily discussed. The main focus of this review involves the synergistic role of electrical and mechanical stimulation, extracellular matrix interactions, and non-cardiomyocyte interactions in facilitating cardiomyocyte maturation. Overall, even with these current natural engineering approaches, pluripotent stem cell-derived cardiomyocytes within three-dimensional engineered heart tissue still remain mostly within the early to late fetal stages of cardiomyocyte maturity. Therefore, although the end goal is to achieve adult phenotypic maturity, more emphasis must be placed on elucidating how the *in vivo* fetal microenvironment drives cardiomyocyte maturation. This information can then be utilized to develop natural engineering approaches that can emulate this fetal microenvironment and thus make prominent progress in pluripotent stem cell-derived maturity toward a more clinically relevant model for cardiac regeneration.

## Introduction

Cardiovascular disease has remained the most common cause of mortality for over a century. According to the most recent report update from the American Heart Association, the total cost of heart disease in the United States from indirect and direct expenses is about $207 billion, with the cost of cardiovascular disease projected to increase over the next 15 years. About 750,000 Americans suffer from a myocardial infarction each year with about 16% of those occurrences leading to mortalities (Mozaffarian et al., 2016).

A myocardial infarction is caused by coronary artery blockage, which leads to substantial necrosis within the heart wall. After a myocardial infarction, a massive inflammatory response occurs to rid the heart wall of all necrotic tissue. Since adult cardiomyocytes are unable to regenerate, deleterious remodeling of the heart wall ensues, which leads to the deposition of dysfunctional fibrotic tissue. Congestive heart failure can then occur as the heart is unable to meet the work load demand (Konstam et al., 2011).

The current gold standard treatment for patients suffering from chronic heart failure is heart transplantation but there are substantial limitations associated with this treatment, such as an inadequate supply and heart rejection. Due to these major limitations, there remains a significant clinical need for a viable cardiac regenerative therapy that can restore myocardial function following a myocardial infarction (Awada et al., 2016). Some promising results have been recently made to induce adult cardiomyocytes within the heart to re-enter the cell cycle by utilizing different biological factors that are associated with cardiomyocyte cell cycle regulation, such as the transcription factor Meis1, microRNAs associated with epigenetic cell cycle modifications, and the hippo pathway yes-associated protein (Eulalio et al., 2012; Mahmoud et al., 2013; Xin et al., 2013). However, even with these recent encouraging results, the induction of enough adult cardiomyocytes to proliferate and restore cardiac function following an MI has largely been a failed effort over the past several decades.

Therefore, many researchers have turned to tissue engineering approaches involving a variety of proliferative cell types in hopes of regenerating the myocardium. Several prominent advances over the course of the past decade have been made with the development of human stem cell-derived three dimensional engineered heart tissue. Many stem cell types have been applied for cardiac regenerative therapy, such as bone marrow and endogenous cardiac stem cells but their respective low cardiac differentiation efficiency and low yield have greatly limited their clinical use (Orlic et al., 2001; Nygren et al., 2004; Pouly et al., 2008). In contrast, pluripotent stem cell-derived cardiomyocytes (PSC-CMs) from embryonic stem cells and induced pluripotent stem cells can both differentiate effectively into cardiomyocytes and remain a promising approach for cardiac regenerative therapy. However, one major obstacle that has prevented the use of either of these PSC-CMs clinically is their apparent immature, fetal-like phenotype (Alcon et al., 2012).

Due to PSC-CM immaturity, researchers have investigated several ways to improve pluripotent stem cell-derived cardiomyocyte maturity, with the most promising results coming from mimicking the normal developmental program that drives cardiomyocytes maturity *in vivo*, known as natural engineering. This review discusses the natural developmental paradigm of *in vivo* CM development as well as the promising *in vitro* natural engineering approaches to drive CM maturity. The main focus of this review is the synergistic role of mechanical and electrical stimulation, extracellular matrix interactions, and non-cardiomyocyte interactions in the facilitation of cardiomyocyte maturation.

## Maturation differences between four cardiomyocyte developmental stages and pluripotent stem cell-derived cardiomyocytes

Pluripotent stem cell-derived cardiomyocytes (PSC-CMs) are significantly immature compared to adult cardiomyocytes (CMs) and exhibit a fetal-like phenotype (Feric and Radisic, 2016). Throughout this review, PSC-CMs will refer to both induced pluripotent stem cell-derived ventricular cardiomyocytes (IPS-CMs) and embryonic stem cell-derived ventricular cardiomyocytes (ESC-CMs).

In order to gain a deeper understanding of this functional and structural immaturity, this section compares the maturation differences between *in vitro* PSC-CMs and four distinct *in vivo* developmental CM stages: early fetal, late fetal, neonatal, and adult CMs. Early fetal CMs relate to the cardiac cells of the heart tube and early cardiac looping stages while late fetal CMs pertain to the late cardiac looping and chamber formation stages. Neonatal CMs refer to CMs present during the first few weeks after birth, in which several significant maturation processes occur, while adult CMs are the fully developed, mature stage. During natural development, CMs dramatically improve their functional and structural maturity, which leads to highly efficient adult CMs. In comparison, *in* vitro methods have ultimately failed at recapitulating the majority of this natural development, and thus have led to the production of PSC-CMs that remain within the early to late fetal CM stages of maturation (Feric and Radisic, 2016).

### Morphology

When comparing PSC-CMs to the developmental CM stages, there exhibits apparent morphological similarities to early fetal CM stages. PSC-CMs and both stages of fetal CMs are morphologically round and single nucleated (Kim et al., 1992; Veerman et al., 2015; Feric and Radisic, 2016). Postnatally, CMs switch to an elongated morphology and some become binucleated (Anversa et al., 1980). Mature adult CMs are elongated and rod-shaped, with about thirty percent of the cells being binucleated (Olivetti et al., 1996; Mollova et al., 2013). The surface area of PSC-CMs and both stages of fetal CMs ranges from about 1,000–1,300 μm^2^ compared to adult CM surface area of about 10,000–14,000 μm^2^ (Li et al., 1996; Ribeiro et al., 2015). As a major transitional period, neonatal CMs increase in surface area with values that are much greater than fetal and PSC-CMs but lower than adult (Anversa et al., 1980). Surface area increases are the result of CMs switching from hyperplastic growth, which involves CM proliferation, to hypertrophic growth, which involves increase in CM size during the neonatal development stages (Bernardo et al., 2010). Neonatal CMs are more aligned than either stage of fetal CMs (Anversa et al., 1980; Feric and Radisic, 2016). Adult CMs are anisotropic, which facilitates electrical conduction and contractility efficiency, compared to the random alignment of PSC-CMs and early fetal CMs (Gerdes et al., 1992; Feric and Radisic, 2016).

### Contractility

There exists substantial differences in the contractile machinery. PSC-CM sarcomeres, the basic contractile unit of muscular tissue, are less organized than late stage fetal CMs (Mummery et al., 2003; Feric and Radisic, 2016). Adult CM sarcomeres are highly aligned and uniformly distributed along cell edges and contain high densities of aligned myofibrillar structures (Maillet et al., 2013; Feric and Radisic, 2016). In comparison, PSC-CMs and early fetal CMs are randomly aligned and distributed, found mostly in the perinuclear region, and contain less dense myofibrillar structures (Chacko, 1976; Mummery et al., 2003; Feric and Radisic, 2016). Late fetal CMs are well organized and aligned while neonatal CMs have even more alignment and a substantial increase in fiber density (Chacko, 1976).

**Figure 1:**
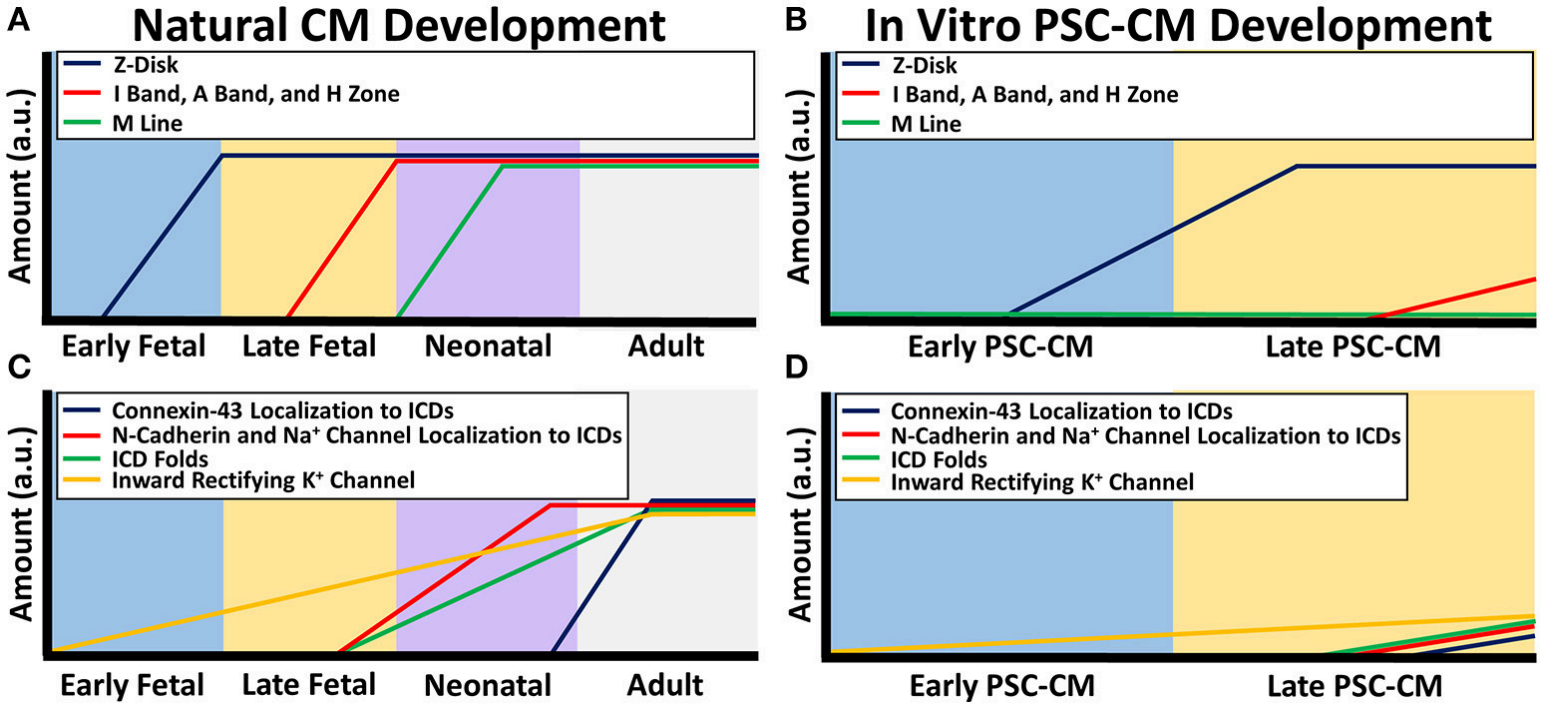
**Sarcomere and electrical coupling differences between natural CM development and PSC-CM development**. The x-axis denotes the CM developmental stage and the y-axis is the amount in arbitrary units (a.u.). **(A)** During natural *in vivo* CM development, sarcomerogenesis occurs with Z-disks forming first in the early fetal stage, followed by I bands, A bands, and H zones in the late fetal stage, and M lines during the neonatal stage. **(B)** During *in vitro* PSC-CM development, early PSC-CMs contain Z-disks but no other distinguishable sarcomeric structures. I bands, A bands, and H zone only become partially distinguishable during later PSC-CM stage but M lines usually are never seen within PSC-CMs. **(C)** Intercalated disk (ICD) development begins during the late fetal CM stage as N-cadherins and sodium channels become more localized to ICDs and apparent ICD folds can begin to be seen. N-cadherin and sodium channel complete ICD localization occurs a few months after birth but connexin-43 complete ICD localization, and thus full ICD maturation, does not occur until a couple years after birth. Inward rectifying potassium channel increases throughout CM development. **(D)** Early PSC-CMs have no localization of N-cadherins, sodium channels, or connexin-43 as well as no distinguishable ICD folds. Some localization to ICDs begins during the late PSC-CM stage but researchers have yet to see full ICD maturity in PSC-CMs. Inward rectifying potassium channel increases slightly from early to late PSC-CM.

Striations are hallmarks of mature sarcomeres formed from overlapping and non-overlapping myofilaments. In adult CMs, all the prominent myofilament regions can be seen including the I bands, A bands, M-line, and H-zone, as well as the Z-disks, which denote the sarcomere ends (Gregorio and Antin, 2000). In contrast, PSC-CM sarcomere striations are mostly indistinguishable. PSC-CMs usually only have Z disks and sometimes I bands since the immature sarcomeres are not organized to provide the different myofilament regions (Figure 1B; Snir et al., 2003). Only one study has been able to produce a small percentage of IPS-CMs with all myofilament regions after 360 days of culturing, but contractile protein expression levels still remained much lower than adult CMs (Kamakura et al., 2013). Early fetal CMs contain only early forming Z disks but late fetal CMs have completely developed Z disks as well as I bands, A bands, and H zones (Figure 1A). M-lines develop during the neonatal period (Chacko, 1976).

Although PSC-CMs have disorganized sarcomeres, the majority of the contractile proteins found in mature CMs can be detected in PSC-CMs, but at much lower levels of expression or present in different isoforms (Veerman et al., 2015). These isoforms change naturally during CM development. The myosin heavy chain structural protein isoform, beta-myosin heavy chain (β-MHC), is prevalent in mature adult ventricular CMs while alpha-myosin heavy chain (α-MHC) is much lower (Reiser et al., 2001). In contrast, both stages of fetal CMs contain both isoforms but α-MHC is more predominant (Razeghi et al., 2001; Xu et al., 2009). After birth, the ratio of β-MHC to α-MHC increases in neonatal CMs to lead to the adult form (Siedner et al., 2003). PSC-CMs have varying amounts of both MHC isoforms due to the heterogeneous culture of various cardiac cell types (Xu et al., 2009; Ivashchenko et al., 2013; Lundy et al., 2013). The myosin light chain (MLC) isoforms change during development. Both stages of fetal CMs contain both isoforms, MLC2a and MLC2v, however, in adult ventricular CMs, only MLC2v can be found (Hailstones et al., 1992; Macera et al., 1992). MLC isoform prevalence switch occurs during the neonatal stage (Chuva de Sousa Lopes et al., 2006). Similar to the MHC isoforms, PSC-CMs contain both isoforms of MLC (Zhang et al., 2013). PSC-CMs likewise exhibit low levels of expression of troponin isoforms, the protein involved in regulating myosin binding to actin, and alpha-actin isoforms, which contributes to the actin myofilaments, compared to adult CMs (Khan et al., 2015; Veerman et al., 2015; Li et al., 2016). Fetal CMs express mostly cTnT1 and cTnT2 which then switch to higher amounts of cTnT3 and cTnT4 during the neonatal stage and is abundant in adult CMs (Siedner et al., 2003). Troponin I isoform changes from slow skeletal troponin I, predominant in fetal and neonatal CMs, to cardiac troponin I found mostly in adult CMs (Siedner et al., 2003). Fetal and neonatal CMs contain mostly skeletal alpha-actin which changes to cardiac alpha-actin within adult CMs (Schwartz et al., 1992).

Due to these differences in protein isoforms and organization, PSC-CMs are unable to contract efficiently, which limits their overall functionality without any further improvements in their maturity. In three-dimensional culture, the active contraction force of PSC-CMs ranges from about 0.1 to 0.5 mN/mm^2^ while adult CMs generate two orders of magnitude larger contractile forces of 10–50 mN/mm^2^ (Mulieri et al., 1992; Holubarsch et al., 1998; Schaaf et al., 2011; Tulloch et al., 2011). Fetal CMs contract with 0.4 mN/mm^2^ forces, which is within the range of PSC-CMs (Ribeiro et al., 2015). Neonatal CMs dramatically increase their contraction strength as the contractility machinery matures, which causes a 2 or 3 fold increase in force compared to the fetal stage (Wiegerinck et al., 2009).

### Electrophysiology

PSC-CMs are known to spontaneously contract asynchronously, due to their immature electrical coupling, while adult CMs are only excited when provided an electrical stimulus and contract in a synchronous manner (Keung et al., 2014). In comparison, early fetal CMs contract very asynchronously and spontaneously during the early stages of heart tube formation. As development progresses, late fetal CMs are more synchronized. Synchronous beating continues in neonatal stages on to adulthood with greater electrical coupling (Liau et al., 2012). Normally, ventricular adult CMs have distinct action potential profiles with four phases. Phase 0 includes sodium and calcium channels, which depolarize the cell. In phase 1, outward potassium channels cause partial cell membrane repolarization. Phase 2 contains a plateau where calcium moves inward and potassium travels outward. This calcium influx leads to excitation-contraction coupling where the cell converts the electrical signal into cross-bridge cycle mediated contractions. Phase 3 ensues immediately after with outward potassium movement bringing the resting membrane potential (RMP) back down to −85 mV. Phase 4 involves the inward rectifying potassium channel (KCNJ2), which maintains the RMP (van den Heuvel et al., 2014).

Similar to their structural machinery, ion channels change throughout cardiomyocyte development, leading to varying action potential profiles and electrophysiological maturity. Unlike adult CMs, PSC-CMs lack a majority of KCNJ2, which causes unstable RMP ranges of −50 to −60 mV (Sartiani et al., 2007; Amin et al., 2010; Ma et al., 2011). Early fetal CMs have minute amounts of KCNJ2, which increases 10 fold in the late fetal CMs and progressively increases into the adult stage (Liu et al., 2016). In correlation with this progressive increase, the RMP of CM stages becomes progressively more negative from −40 mV in early fetal stages to −85 mV in adult stages (Gennser and Nilsson, 1970; Mummery et al., 2003). PSC-CMs have pacemaker current channels, which are absent or expressed in low amounts in adult ventricular CMs (Sartiani et al., 2007; Verkerk et al., 2007; Lieu et al., 2013). Similarly, early fetal CMs express high levels of pacemaker channels, which decrease during the late fetal and neonatal stages (Liu et al., 2016). Pacemaker channels along with unstable RMPs are responsible for the spontaneous contractility of PSC-CMs and early fetal CMs (Veerman et al., 2015). T-type calcium channels, that are prominent within cardiac conducting cells, are not present within adult ventricular CMs but are detected in varying amounts within PSC-CMs most likely due to their heterogeneous differentiation (Bkaily et al., 2005; Ono and Iijima, 2010). During development, early fetal CMs express low T-type calcium channel amounts which decreases in late fetal and is basically absent by the neonatal stage of CM development (van den Heuvel et al., 2014). In contrast, L-type calcium channels increase progressively from the early fetal stage into the adult stage. PSC-CMs generally have lower amounts of L-type calcium channels within the fetal range (Liu et al., 2016).

Underdeveloped cell-cell connections between PSC-CMs similarly impact their electrophysiological function (Figure 1D). Cardiac specific cell-cell connections, called intercalated discs (ICDs), contain adheren junctions, desmosomes, gap junctions, and sodium channels, which allow rapid transmission of electrical signals and contractile forces within CMs (Noorman et al., 2009; Wang et al., 2012). Connexin-43, N-cadherin, SCN5A are respectively the most prevalent gap junction, adheren junction proteins, and sodium channels, and are found within adult, fetal, neonatal, and PSC-CMs at similar amounts (Chen et al., 1994; Noorman et al., 2009; Lundy et al., 2013; Liu et al., 2016). However, spatiotemporal distribution of these structures changes during development (Figure 1C). Adult CMs have them densely distributed around mature ICDs (Angst et al., 1997; Jansen et al., 2010; Feric and Radisic, 2016). Early fetal CMs and PSC-CMs contain these proteins sporadically distributed along the cell membrane with no distinguishable intercalated discs present, which greatly contributes to their immature asynchronous beating behavior (Vreeker et al., 2014; Feric and Radisic, 2016). Late fetal CMs have some localization of N-cadherin and sodium channels to their premature ICD but sporadic connexin-43 distribution. Sodium channels and N-cadherins are co-localized with each other on ICDs at 5 months postnatal development while connexin-43 is not completely localized to ICDs until 7 years (Vreeker et al., 2014). Neonatal ICDs have a less folded morphology with a smaller width compared to adult ICDs (Wilson et al., 2014).

The immature electrophysiology of fetal CMs and PSC-CMs directly affects several electrical efficiency parameters. The depolarization velocity of fetal and PSC-CMs ranges from a 6 to 50 fold decreased amount compared to adult CMs (Mummery et al., 2003; Koncz et al., 2011; Doss et al., 2012; Ribeiro et al., 2015; Feric and Radisic, 2016). The capacitance, which is the ionic charge that is necessary to fluctuate the potential of the cell membrane, is generally lower in PSC-CMs and fetal CMs compared to adult and neonatal CMs (Drouin et al., 1995; Zhu et al., 2009; Polak and Fijorek, 2012; Sheng et al., 2012; Feric and Radisic, 2016). The velocity of the action potential propagation through CMs, known as conduction velocity, is lower in early PSC-CMs than fetal CMs (Gennser and Nilsson, 1970; Taggart et al., 2000; Lee et al., 2012; Veerman et al., 2015). Conduction velocity naturally increases during CM development (Gennser and Nilsson, 1970; Yang et al., 2014).

### Calcium handling

**Figure 2:**
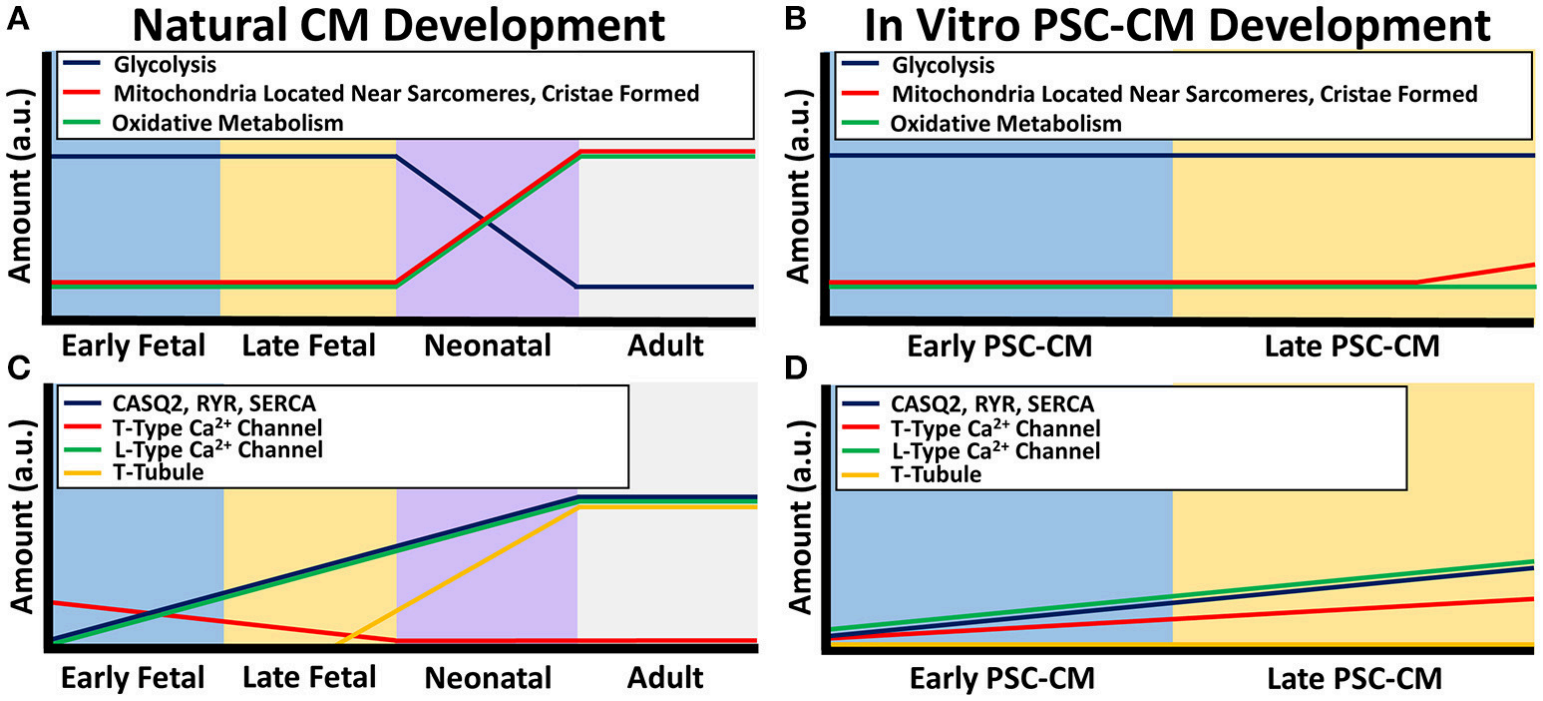
**Calcium handling and metabolism differences between natural CM development and PSC-CM development**. The x-axis denotes the CM developmental stage and the y-axis is the amount in arbitrary units (a.u.). **(A)** Metabolic maturity begins during the neonatal CM stage as the energy production switches from mostly glycolysis to oxidative metabolism along with an increase in microchondria localization to developing sarcomeres and mitochondrial cristae formation. **(B)** PSC-CM metabolic development remains mostly within the early to late fetal CM stage. Some cristae have shown to form in late PSC-CM mitochondria as well as some localization of mitochondria to developing sarcomeres. **(C)** Calcium handling proteins calsequestrin (CASQ2), ryanodine receptors (RYR), sarcoplasmic endoplasmic reticulum calcium ion ATPase (SERCA), and L-type calcium channels gradually increase from early fetal to the neonatal CM stage. Transverse tubules (T-tubules) begin to form in the late fetal CM stage and fully form in the neonatal CM stage. T-type calcium channels decreases during the fetal stages until mostly not present in the neonatal CM stage. **(D)** In PSC-CMs, CASQ2, RYR, SERC, L-type calcium channels, and T-type calcium channels gradually increase from the early PSC-CM stage to the late PSC-CM stage. However, T-tubules have mostly never formed in any PSC-CM stages.

Structures involved in calcium handling likewise mature throughout development (Figure 2C). Normally in adult CMs, action potentials propagate down transverse tubules, which are deep protrusions of the sarcolemma that permit efficient action potential propagation. These action potentials cause activation of L-type calcium channels along the transverse tubules, which leads to calcium induced calcium release from the sarcoplasmic reticulum (SR) by ryanodine receptors (RyR) that are co-localized with the L-type calcium channels. The sarcoplasmic reticulum stores significant amounts of calcium through the use of calcium handling buffer proteins called calsequestrin (CASQ2). This intracellular calcium release leads to excitation-contraction coupling as the myofilaments undergo cross-bridge cycles to contract. After the contractions are complete, intracellular calcium is taken back up into the sarcoplasmic reticulum by a specialized structure called the sarcoplasmic endoplasmic reticulum calcium ion ATPase (SERCA). Calcium is transported out of the cell by the sodium calcium exchanger (NCX) (van den Heuvel et al., 2014).

Conversely, PSC-CMs have underdeveloped calcium handling protein structures (Figure 2D). They do not have transverse tubules, which significantly effects the efficiency of the calcium handling and excitation-contraction coupling (Snir et al., 2003; Satin et al., 2008). PSC-CMs have underdeveloped SR with generally low amounts of SERCA, RyR, and CASQ2 (Dolnikov et al., 2006; Binah et al., 2007; Satin et al., 2008; Lee et al., 2011; Synnergren et al., 2011; van den Heuvel et al., 2014). The calcium handling maturity of PSC-CMs are similar to early fetal CMs. Transverse tubules are absent in early fetal CMs and present as only small indentations of the sarcolemma in late fetal CMs (Ziman et al., 2010). During the neonatal stage, transverse tubules become fully developed by about 2–3 weeks (Liau et al., 2012). In terms of calcium kinetics, since fetal CMs and PSC-CMs have lower amounts of CASQ2, RyR, SERCA, and L-type calcium channels, the immature calcium handling relies more heavily on sarcolemma calcium inflow most likely from T-type calcium channels and the NCX as well as small amounts of calcium release from the SR by IP3 regulated calcium channels (van den Heuvel et al., 2014). Similar to other structures, the maturation of calcium handling occurs during the neonatal stage and therefore increases with development (Ziman et al., 2010).

### Metabolism

Dissimilarities in energy production are prevalent between native CMs and PSC-CMs (Figure 2B). Adult CMs have a mature network of ovular-shaped mitochondria that are approximately 35% of the cellular volume and are aligned in the direction of the sarcomeres to provide ample ATP for contraction (Kim et al., 1994; García-Pérez et al., 2008; Piquereau et al., 2010, 2013). PSC-CMs have immature mitochondria that are disorganized around the perinuclear region, present in smaller amounts and sizes (St. John et al., 2005; Robertson et al., 2013; Keung et al., 2014). Cristae, which are folds that give mitochondria a significant surface area for efficient cellular respiration, are absent within PSC-CMs but densely packed within adult CMs (Porter et al., 2011; Feric and Radisic, 2016). In adult CMs, the majority of energy production occurs by oxidative metabolism while PSC-CMs use mostly glycolysis, which is less efficient (Lopaschuk and Jaswal, 2010; Rana et al., 2012; Keung et al., 2014).

From a developmental standpoint, maturation of the metabolism occurs after birth when cardiomyocytes are exposed to higher demands of energy as well as oxygen and fatty acids (Lopaschuk and Jaswal, 2010; Keung et al., 2014). Fetal CMs contain small, cristae absent, rounded mitochondria that are not localized adjacent to sarcomeres and rely on glycolysis for energy production (Porter et al., 2011). After birth, the metabolic stress causes neonatal CMs within 7 days to switch to mostly oxidative metabolism with an increase in mitochondria size and amount, development of cristae, and a more ovular-shaped mitochondria morphology (Figure 2A; Yatscoff et al., 2008; Kreipke et al., 2016). Besides the role in energy production, metabolism regulates cellular processes. Therefore, the switch to a high metabolic stress may be one of the driving factors of the significant maturation that occurs during the neonatal stage (Yanes et al., 2010; Folmes et al., 2011).

### Proliferation ability

The CM developmental stages show drastic differences in their ability to proliferate. Early fetal CMs are highly proliferative but start to decrease rapidly into the late fetal CM stage. Postnatally, neonatal CMs have a window of proliferative ability that lasts for about 7 days before their cell cycle machinery is silenced permanently leading to non-proliferative adult CMs (Ikenishi et al., 2012). PSC-CMs overall are mostly capable of proliferating (Feric and Radisic, 2016). Surprisingly, neonatal mouse hearts can regenerate following a partial tissue removal but this ability diminishes after the first 7 days post birth (Porrello et al., 2011). Studies have shown that oxygen exposure reduces neonatal CM proliferative ability and therefore the change to a mostly aerobic environment post birth could be partially responsible for this shift (Puente et al., 2014). Other researchers hypothesize that metabolic stress similarly plays a key role in this proliferation change (Yutzey, 2014). Overall, however, the permanent cell cycle arrest seems to be necessary for CM maturation and thus heart function (Takeuchi, 2014). As evidence, rare cases of mitogenic cardiomyopathy in human infants have been reported where the neonatal CMs have atypical proliferative ability but an immature phenotype that results in lethality due to cardiac dysfunction (Chang et al., 2010; Shenje et al., 2014).

### Current gap in PSC-CM maturity

**Figure 3:**
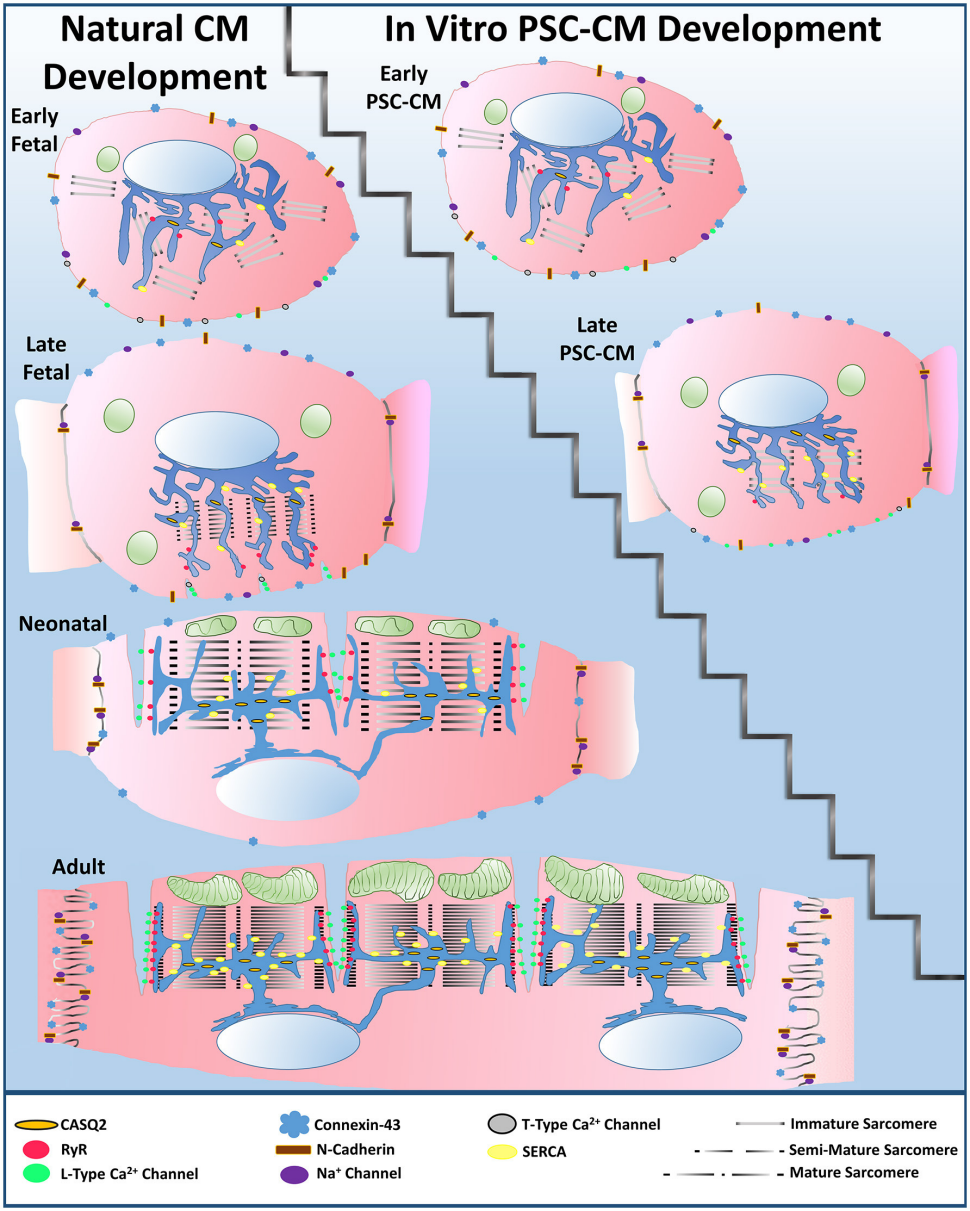
**Large divide between natural cardiomyocyte development and *in vitro* PSC-CM development**. PSC-CMs generally remain within the early to late fetal CM stages even after applying natural engineering approaches, such as mechanical stimulation, electrical stimulation, non-cardiomyocyte interactions, or extracellular matrix interactions to improve their overall maturity to a late PSC-CM stage.

In summary, a large divide in native CM development vs. PSC-CM development is apparent (Figure 3). Human PSC-CMs within three-dimensional culture systems have ultimately failed to obtain all characteristics of late fetal CM maturity, even after improving their maturity through natural engineering approaches. Most achievements in PSC-CM maturity occur at a single cell level while syncytial units of uniformly more mature PSC-CMs have not been developed well in three-dimensional engineered tissues. As evidence, unlike late fetal CMs, no human PSC-CMs have been observed to have early indentations of developing transverse tubules (Satin et al., 2008; Ziman et al., 2010). The majority of sarcomeres within late PSC-CM tissue constructs likewise do not have almost completely organized sarcomeres with Z-disks, A bands, I bands, and H zones (Chacko, 1976; Snir et al., 2003). KCNJ2 expression in PSC-CMs also still remains mostly lower than late fetal CMs (Amin et al., 2010; Liu et al., 2016). In addition, when native CMs are isolated and placed within *in vitro* culture they have been observed to go backwards in maturity (Nguyen et al., 2015). When more mature native CMs are cultured *in vitro*, they can mature more than PSC-CMs, such as forming fully developed transverse tubules (Bian et al., 2014a). Therefore, more emphasis needs to be placed on understanding the natural fetal stages of CM development. Once researchers understand how to more accurately recapitulate the fetal stages of CM development, further progress in PSC-CM maturity can be made toward the neonatal stage and thus eventually to the adult mature phenotype.

## Natural developmental program that drives *in vivo* cardiomyocyte maturation

**Figure 4:**
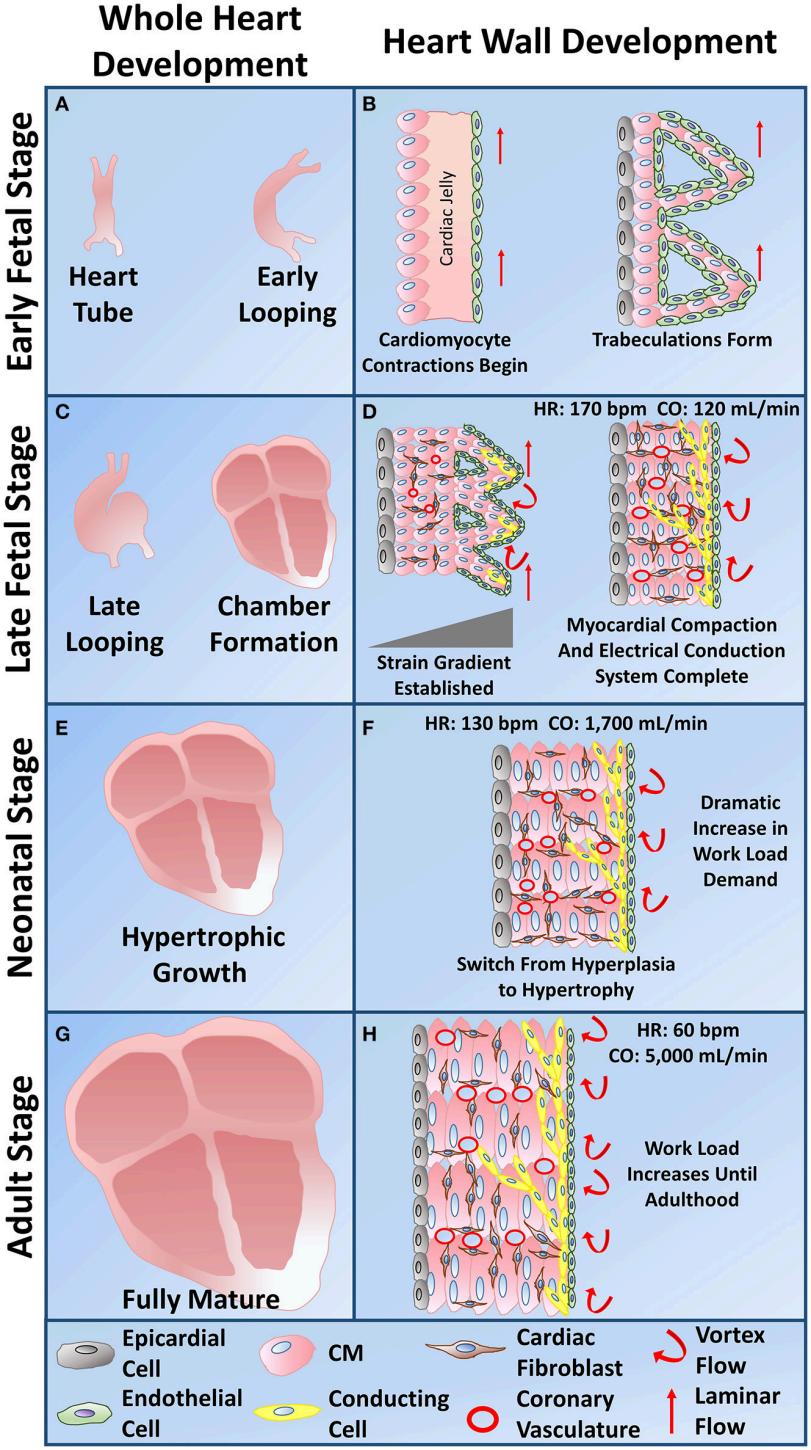
**Whole heart and heart wall development schematic. (A)** The early fetal CM stage refers to the formation of the heart tube and early cardiac looping. **(B)** Within the heart tube, early fetal CMs line the outer edge of the heart wall and begin to spontaneously beat, a cardiac jelly consisting of early cardiac ECM lies within the middle layer, and an epithelial layer of cells lines the inner layer. During early cardiac looping, trabeculations begin to form where the cardiomyocytes form protrusions to increase their surface area in the absence of coronary vasculature. Epicardial-derived progenitor cells also form an early epicardial outside layer of the heart wall. Hemodynamic flow remains linear during the early fetal CM stage. **(C)** The late fetal CM stage consists of the late cardiac looping phase as well as the formation of the four cardiac chambers. **(D)** The late cardiac looping phase consists of early compaction of the myocardium as well as epicardial-derived progenitor cells undergoing epithelial to mesenchymal transition and migrating into the myocardium to differentiate into cardiac fibroblasts. Early coronary vasculature begins to develop as myocardial compaction begins. Also hemodynamic vortex flow begins to occur and early specification of cardiac conducting cells begins to take place on the inner myocardial wall layer within the trabeculae. The chamber formation stage consists of complete myocardial compaction as coronary vasculature becomes fully developed. Cardiac fibroblasts surround all cardiomyocytes and the cardiac conduction system, known as the His-Purkinje fibers, becomes fully developed to form branching networks residing underneath the endothelial layer. Hemodynamic vortex flow becomes consistent during chamber formation. **(E)** The neonatal stage is known for its change in growth from hyperplasia to hypertrophy as the heart grows in size to meet the work load demand. **(F)** At the neonatal stage, cardiomyocytes undergo hypertrophy as a dramatic increase in work load demand ensues leading to an increase in cardiomyocyte maturation. **(G)** The adult stage contains a fully mature heart with an increase in thickness of the left ventricular wall. **(H)** The cardiomyocytes within the heart wall during the adult stage are fully mature aligned cells that have high functional efficiency to meet the work load demand of the fully grown body system.

During the natural developmental program *in vivo*, mechanical stimuli, electrical stimuli, extracellular matrix interactions, and non-cardiomyocyte interactions synergistically combine in a spatiotemporal manner to effectively drive CM maturity from the immature early fetal CM stage to the functionally mature adult CM stage. Early heart formation involves a heart tube that consists of a myocardial and endocardial layer with a “cardiac jelly” extracellular matrix layer in between. During early cardiac looping, trabeculations start to form in the absence of any coronary vasculature. Compaction then follows during the late cardiac looping stage and chamber formation stage as the coronary vasculature develops. The heart then undergoes hypertrophic growth within the neonatal to adult stages (Sedmera et al., 2000). All of the natural developmental influences must combine in the correct manner for the heart to develop into an efficient four chamber pumping mechanism (Figure 4). Subsequent sections explain the importance of the four natural influences on the developmental program of CMs *in vivo*.

### Natural development program of *in vivo* mechanical stimuli

Mechanical stimuli affect all developmental stages of the heart from early embryogenesis to post-natal maturation (Takahashi et al., 2013). As a whole organ, the heart's main function is a mechanical pump that consistently responds to mechanical stimuli throughout its entire lifetime (Jacot et al., 2010; Takahashi et al., 2013). The influence of mechanical stimuli on the heart became apparent over 100 years ago with the establishment of the Frank-Starling Law, which indicates that as the heart tissue stretches from filling of blood, the heart's contractile force also increases (Konhilas et al., 2002). Studies on zebrafish embryos have shown that a lack of proper mechanical load can lead to abnormal heart development, which provides further evidence for the importance of mechanical stimuli during development (Hove et al., 2003).

One important concept of the heart involves the mechanoelectric feedback (MEF), where there exists an interplay between mechanical input and electrical output. MEF regulates the beating rate as well as excitation-contraction coupling within cardiomyocytes (Kohl and Ravens, 2003). This feedback mechanism involves the many mechanosensitive ion channels that respond to mechanical stimuli (Hu and Sachs, 1997; Takahashi et al., 2013). Studies on embryonic chick heart development have shown that hemodynamic loads directly influence maturation of the heart's conduction system (Figure 4; Reckova et al., 2003). Mechanical stretch leads to an increase in connexin-43, and thus an increase in the overall conduction velocity (Sheehy et al., 2012). Therefore, mechanical stimuli have a direct influence on the electrophysiological maturation of CMs.

Cardiomyocytes experience three main types of constant mechanical stimuli that influence development: stretch during heart filling, hemodynamic loads from blood flow, and active self-induced cytoskeletal contractions during heart ejection (Taber, 2001). As the developmental stages of CMs progresses, more mechanical stimuli play an influential role. Early fetal CMs begin to contract spontaneously and asynchronously in the early heart tube before coordinated blood flow and the sarcomeres completely develop (Lindsey et al., 2014; Geach et al., 2015). Although the reasoning behind this early spontaneous beating is still being investigated, studies have shown that the early contractions cause hemodynamic forces to develop, which in turn impact subsequent heart development (Granados-Riveron and Brook, 2012). A study on frog heart development showed that early CM contractions drive sarcomerogenesis (Geach et al., 2015). Therefore, the contractions may act as a feedback loop to continue early fetal CM development within the heart tube to the next developmental stage.

The cross-bridge cycles of the early fetal CM's cytoskeleton are known to drive morphogenesis of the cardiac looping phase by causing the heart tube to bend (Manning and McLachlan, 1990; Taber, 2001). In contrast, extrinsic mechanical stimuli, such as an increase in hemodynamic force, are what cause the cardiac loop to twist and rotate (Wyczalkowski et al., 2012). During the late fetal CM stage of heart chamber formation involving trabeculation and compaction of the myocardium, strain begins to play a larger role (Figure 4). A strain gradient, which varies across the myocardial wall with higher amounts of strain in the inner trabeculated layer, may influence late fetal CM maturation (Damon et al., 2009). The outer compact layer contains late fetal CMs that have a greater mitotic activity than late fetal CMs found within the trabeculated layer. Therefore, an increase in strain causes a reduction in proliferation rate and thus may drive CM developmental maturity (Sedmera and Thompson, 2011). Other studies on the impact of hemodynamic forces have shown that an increase in hemodynamic blood flow causes ventricular CMs to become more elongated and rod-shaped (Auman et al., 2007). Compaction increases during the late cardiac looping and chamber formation stages of heart development due to the change in hemodynamic flow from laminar to vortexed flow (Figure 4; Samsa et al., 2013).

As the demand for more blood increases throughout development, an increase in hemodynamic loading on cardiomyocytes occurs. CMs become more mature to meet these higher workload demands, especially during the neonatal to adult stages (Figure 4; Taber, 2001; Zhu et al., 2014). As a result, cardiac output, which has only been measured during the four chamber heart stage, increases exponentially throughout development with values of 120 mL/min at 15–20 weeks gestation, 1,700 mL/min around the neonatal stage, and around 5,000 mL/min in adulthood (Figure 4; De Smedt et al., 1987; Matthews et al., 2012). Human heart rate varies greatly during development. From the early heart tube stage to the four chambered heart stage, heart rate progressively increases to about 170 bpm, which then declines from the late fetal stage into the neonatal stage to a value around 130 bpm (Figure 4; Doubilet and Benson, 1995; Hornberger and Sahn, 2007; Lindsey et al., 2014). From the neonatal to adult stage, heart rate continues to decrease until reaching about 60 bpm (Nunes et al., 2013b).

Besides mechanosensitive ion channels, several other protein structures are involved in mechanotransduction pathways of cardiomyocytes. These protein structures include the cytoskeleton, focal adhesions, integrins, rho kinase, integrin-linked kinase, and extracellular signal-related kinase (Takahashi et al., 2013). Mechanical stimuli causes rho/rock activation which regulates several cellular processes, such as hypertrophy, proliferation, differentiation, and maturation (Loirand et al., 2006). Thus, mechanical stimuli are crucial to the overall natural developmental program of CMs.

### Natural development program of *in vivo* electrical stimuli

Electrical conduction is also a vital part of cardiac functionality. Studies on zebrafish hearts have shown that impaired cardiac conduction leads to abnormal cardiac remodeling and development (Chi et al., 2010). Development of the cardiac electrical conduction system has been readily studied in chick model organisms. During the early tubular heart stage, a slow and linear electrical conduction is established. Therefore, CMs undergo a constant electrical stimuli from the early onset of CM specification. As the heart tube undergoes the cardiac looping phase, the conduction velocity increases but the conduction is still in the blood flow direction. As the myocardial trabeculations form, temporary conduction pathways develop in the anterior and posterior. These temporary conduction pathways are present only before the development of the His-Purkinje fiber network, which denotes a mature conduction system. The trabeculations are also involved in the early conduction system of the heart since they can electrically conduct well. As the four chambers of the heart form, the direction of conduction from base to apex shifts to a mature apex to base path. This shift occurs when the His-Purkinje fibers, composed of specialized cardiac conducting cells, have fully formed underneath the endothelial layer, which allows coordinated contractions from the apex that push blood out of the ventricles (Sedmera et al., 2004). Therefore, the late fetal CMs experience more coordinated electrical impulses that further drive their structural and functional maturity. The functional maturity of the electrical conduction system is complete by the end of embryonic cardiac development. However, further maturation of CM electrophysiology occurs after birth. As transverse tubules develop during the neonatal stage, the excitation-contraction coupling of CMs mature, which allow action potentials to reach deep within the CMs (Ziman et al., 2010).

Electrical stimuli directly impact the electrophysiological function of CMs. Gap junctions, which regulate the propagation of electrical signals between neighboring CMs are critical to the electrical conduction (Angst et al., 1997). Several studies using *in vivo* connexin protein knockouts have shown to lead to CM electrical conduction defects, such as arrhythmias (Britz-Cunningham et al., 1995; Simon et al., 1998). The ion channels are also critical structures. Mutations in ion channels can lead to pathological disease states, such as mutations in voltage gated potassium channels, which cause ventricular tachycardia (Xiong et al., 2015). As CMs undergo more coordinated and efficient electrical stimuli with the development of the mature cardiac conduction system, the CMs respond to their environment by upregulating electrophysiological structures. Studies on electrical stimuli of CMs have shown an increase in cell size, alignment, connexin-43 expression, ion channel expression, and sarcomere organization (Baumgartner et al., 2014). Overall, efficient propagation of electrical signals is a crucial aspect of CM natural developmental program and functionality.

### Natural development program of *in vivo* extracellular matrix interactions

The extracellular matrix (ECM) plays a dynamic role in the natural development of each CM stage as it provides structural support, modulates many cellular processes, and is constantly being remodeled in response to various stimuli (Lockhart et al., 2011). Since the ECM helps withstand the continuous mechanical stimuli associated with the never ending cardiac cycles throughout an organisms lifetime, it is a crucial component of the heart (Mishra et al., 2013). Deviations from the normal ECM can cause several cardiac formation abnormalities as shown in studies on *in vivo* mouse heart development (Lockhart et al., 2011). Congestive heart failure occurs due to adverse ECM remodeling following a myocardial infarction and therefore undesirable changes in the ECM are linked to several disease states (Konstam et al., 2011). The ECM is also associated with several mechanotransduction pathways through integrin-ECM connections that drive cellular processes (Corda et al., 2000). In animals that can regenerate their myocardium, such as newts, the ECM deposition in a trabecular network fashion is a critical first step of cardiac regeneration (Piatkowski et al., 2013).

During cardiac development, the ECM drastically changes its composition and organization to lead to the formation of the complex four chambered heart. The most significant changes occur during the neonatal stage when the ECM is rapidly remodeled to resemble the adult myocardium composition within 2–3 weeks post birth. From the neonatal and adult stages, the amount of ECM deposition increases (Sullivan and Black, 2013). The most functionally relevant cardiac ECM proteins are collagen type I and III for structural support, basement membrane collagen type IV for facilitating cellular alignment, fibronectin which connects integrins to other ECM proteins, and elastin to provide elastic properties (Hanson et al., 2013). Collagen proteins increase in structural organization and amount as the heart develops to meet the higher work load demands (Gershlak et al., 2013). No distinct structural collagen fibers are found during the early fetal stage. However, as development progresses, collagen fiber networks can be found in late fetal CMs, which increase organization and density even more during the neonatal stage (Hanson et al., 2013). Fibronectin is essential to heart development. If absent within mouse embryos, inadequate heart tube formation occurs (George et al., 1993). The role of fibronectin in early cardiac morphogenesis has been known for almost 30 years. If fibronectin is blocked, cardiac precursor cells cannot migrate appropriately (Linask and Lash, 1988). Similar to collagen, the organization of fibronectin increases significantly during the neonatal stage but the amount of fibronectin is higher in both fetal stages (Sheehy et al., 2012; Hanson et al., 2013). Basement membrane proteins, such as collagen type IV, laminin, glycoproteins and proteoglycans likewise can be found in all stages but increases in organization and density during the neonatal stage and surrounds CMs of the adult stage (Hanson et al., 2013; Yang et al., 2015). Studies have shown that laminin organization facilitates sarcomere organization throughout the neonatal stage of CMs (Yang et al., 2015). Elastin remains mostly constant during the stages of CM development (Hanson et al., 2013).

Other proteins are found throughout the ECM and impact CM development. Hyaluronan, a GAG protein, is known to facilitate proliferation and is involved in cardiac morphogenesis (Baldwin et al., 1994; Toole, 2001). Perlecan and versican, which are proteoglycans, are involved in cardiac morphogenesis since disruption of these proteoglycans causes cardiac formation abnormalities (Henderson and Copp, 1998; Costell et al., 2002; Lockhart et al., 2011). Matrix metalloproteases regulate ECM remodeling and modulate CM contraction and calcium handling (Mishra et al., 2013).

Besides the overall ECM composition, stiffness is a critical factor that influences CM behavior. ECM stiffness has many effects on CMs, such as contractility, calcium handling, and cytoskeletal structure (Takahashi et al., 2013). A study on chick heart development demonstrated that stiffness differences along the heart tube facilitated the heart looping process (Zamir et al., 2003). During the neonatal CM stage after birth, stiffness of the heart wall is known to significantly increase between 2 and 3 fold and may be due to the increase in the neonatal ECM organization (Jacot et al., 2010; Gershlak et al., 2013). Understanding how the ECM interacts with CMs *in vivo* will enable researchers the ability to naturally engineer heart tissue that more closely mimics physiological conditions for enhanced maturity.

### Natural development program of *in vivo* non-cardiomyocyte interactions

Non-cardiomyocytes readily interact with cardiomyocytes and influence cardiac development through a variety of different mechanisms. From a developmental perspective, non-cardiomyocytes are required for the specification of the cardiac lineage. Early on during development, the anterior endoderm induces the specification of the cardiac mesoderm, by FGF8 signaling, which differentiate into all myocardial cell types (Schultheiss et al., 1995; Alsan and Schultheiss, 2002). Several types of cells reside within the ventricular heart wall including cardiomyocytes, cardiac fibroblasts, vascular smooth muscle cells, and endothelial cells. CMs directly interact with endothelial cells derived from the endocardium, as well as cardiac fibroblasts derived from the epicardium (Brutsaert, 2003; Sullivan and Black, 2013). The only stage of CMs that has minimal interactions with non-CMs are the early fetal CMs that reside within the heart tube and early looping stage.

#### Epicardial-derived cells

Although cardiomyocytes occupy the majority of the myocardium, they constitute only about 30% of the total heart cells (Bergmann et al., 2015). Cardiac fibroblasts (CFs) are the other major cell type that is present throughout the myocardium and readily interacts with CMs (Zhang et al., 2012).

During the middle of the cardiac looping stage of cardiac development, epicardial-derived progenitor cells (EPDCs) undergo epithelial to mesenchymal transition, migrate into the developing myocardium, and differentiate into cardiac fibroblasts (CFs) (Figure 4; Sullivan and Black, 2013). Therefore, early fetal CMs do not interact directly with any CFs. CFs have extended spindle-like morphologies and are known for modulating the cardiac ECM and thus play an influential role in providing structural integrity to the heart (Baudino et al., 2006). As the work load increases, the CFs begin depositing more ECM to provide structural support. After birth, ECM remodeling accelerates as CFs degrade the fetal ECM and deposit a variety of proteins including collagen type I, II, and V, as well as fibronectin and vitronectin. Further growth then occurs by CM hypertrophy and increasing deposition of cardiac ECM (Sullivan and Black, 2013). Turnover of the cardiac ECM occurs throughout the organism's lifetime, with studies demonstrating collagen turnover rates of 80–120 days (Shirwany and Weber, 2006).

This cardiac ECM turnover by CFs exists as a fine balance between production and degradation. If altered, pathological remodeling can ensue. Following a myocardial infarction, CFs are prompted to change into a proliferating myofibroblast and produce ECM to replace necrotic tissue, which becomes a fibrotic network (Baudino et al., 2006; Sullivan and Black, 2013). As the fibrotic infarcted region's stiffness increases, CFs are triggered to continue producing more ECM, leading to a deleterious adverse remodeling feedback loop (Sullivan and Black, 2013).

Besides CF's involvement in overall cardiac ECM remodeling, CMs and CFs regulate one another by cross talking through paracrine signaling, cell-cell direct connections, and ECM indirect interactions. Both cell types express many different signals which include transforming growth factor-beta, endothelin-1, fibroblast growth factor 2, interleukins, vascular endothelial growth factor, angiotensin II, and tumor necrosis factor-alpha that modulate cellular processes (Zhang et al., 2012). Interleukin-6 amplifies CM proliferation and hypertrophy and is only highly expressed when CMs and CFs are directly interacting with one another, which indicates the importance of these cell-cell interactions (Sarkar et al., 2004; Ottaviano and Yee, 2011). CFs can also affect the electrophysiological function of CMs by paracrine signals that modulate CM ion channels and gap junctions (Merle et al., 1995; Doble et al., 1996; Goette and Lendeckel, 2008). CFs are unable to be electrically stimulated but when connected to CMs via gap junctions they can pass current. The proportion of CFs to CMs as well as the strengths of these connections are important parameters in terms of cardiac electrophysiological function (Zhang et al., 2012). Adheren junctions also connect CMs to CFs which can allow for mechanical stimuli to be directly transmitted and thus activate mechanotransduction processes (Thompson et al., 2011).

EPDCs themselves also drive developmental processes within the myocardial wall (Lie-Venema et al., 2007). One study demonstrated that an absence of EPDCs in chick and mouse models led to a significant reduction in N-cadherin and connexin-43 within CMs of the myocardium (Weeke-Klimp et al., 2010). Epicardial layer defects have also shown to lead to myocardial compaction and cardiac looping abnormalities (Eralp et al., 2005; Merki et al., 2005; Takahashi et al., 2014). After a myocardial injury, the epicardium secretes paracrine factors and fibronectin to facilitate myocardial protection in zebrafish hearts, which have the capability to regenerate their hearts, indicating the importance of the epicardium (Wang et al., 2013).

#### Endocardial-derived cells

Extensive evidence points to the significance of endothelial cell interactions with cardiomyocytes for cardiac development and functionality. Endothelial cells that are present within the heart come from two classes: (1) vascular endothelial cells that compose the coronary vasculature and are derived from the epicardium and (2) cardiac endothelial cells that make up the myocardial capillaries and endocardium. Unlike vascular endothelial cells, cardiac endothelial cells (CEs) are in direct contact with cardiomyocytes. CEs influence cardiomyocyte metabolic activity, contractility, hypertrophy, and overall beating rhythm through paracrine signaling (Brutsaert, 2003). Cardiac endothelial cells are present during the heart tube stage (Figure 4; Sedmera et al., 2000).

Several studies on zebrafish and mouse heart development have shown that the absence of the endocardium or disruption in the functional interactions between the endocardium and myocardium leads to several myocardial development abnormalities. Mutations in CE secreted factors, such as VEGF, neuregulin growth factor, serotonin, and angiopoietin within *in vivo* models cause myocardial defects, such as a reduction or loss of trabeculations, a crucial step in development of the myocardium (Fong et al., 1995; Lee et al., 1995; Kramer et al., 1996; Suri et al., 1996; Haigh et al., 2000; Nebigil et al., 2000). Endothelial cells increase the survivability of CMs by secretion of several other factors such as nitric oxide (Leucker et al., 2013).

Less studies have been performed on understanding CE induced maturation of CMs. However, factors such as nitric oxide, endothelin-1, angiotensin II, and prostaglandin I2 might facilitate CM hypertrophy (Brutsaert, 2003). CEs regulate contractility of neighboring CMs through a variety of signals, which includes nitric oxide and endothelin-1 (Smith et al., 1991; Ramaciotti et al., 1992; Rich and McLaughlin, 2003; Champion et al., 2004). CEs impact CM electrophysiology since disruptions in endothelial cells lead to arrhythmic cardiac beating (Hassanabad et al., 1998). In summary, further studies need to be conducted to fully elucidate the intricate role of CM maturation driven by non-cardiomyocytes.

## Natural engineering *in vitro* approaches to drive cardiomyocyte maturation

The functional immaturity of PSC-CMs greatly limits their ability to be used clinically for cardiac regeneration. As evidence, one recent study transplanted human IPS-derived cardiac progenitor cells and human IPS-CMs into neonatal rat hearts to assess if the *in vivo* environment improves their functional maturity. Results showed the cells were only able to partially mature after 3 months *in vivo* indicating the necessity to first mature the PSC-CMs *in vitro* (Kadota et al., 2017). IPS cells have also shown to retain the epigenetic age of the donor (Sardo et al., 2016).

**Figure 5:**
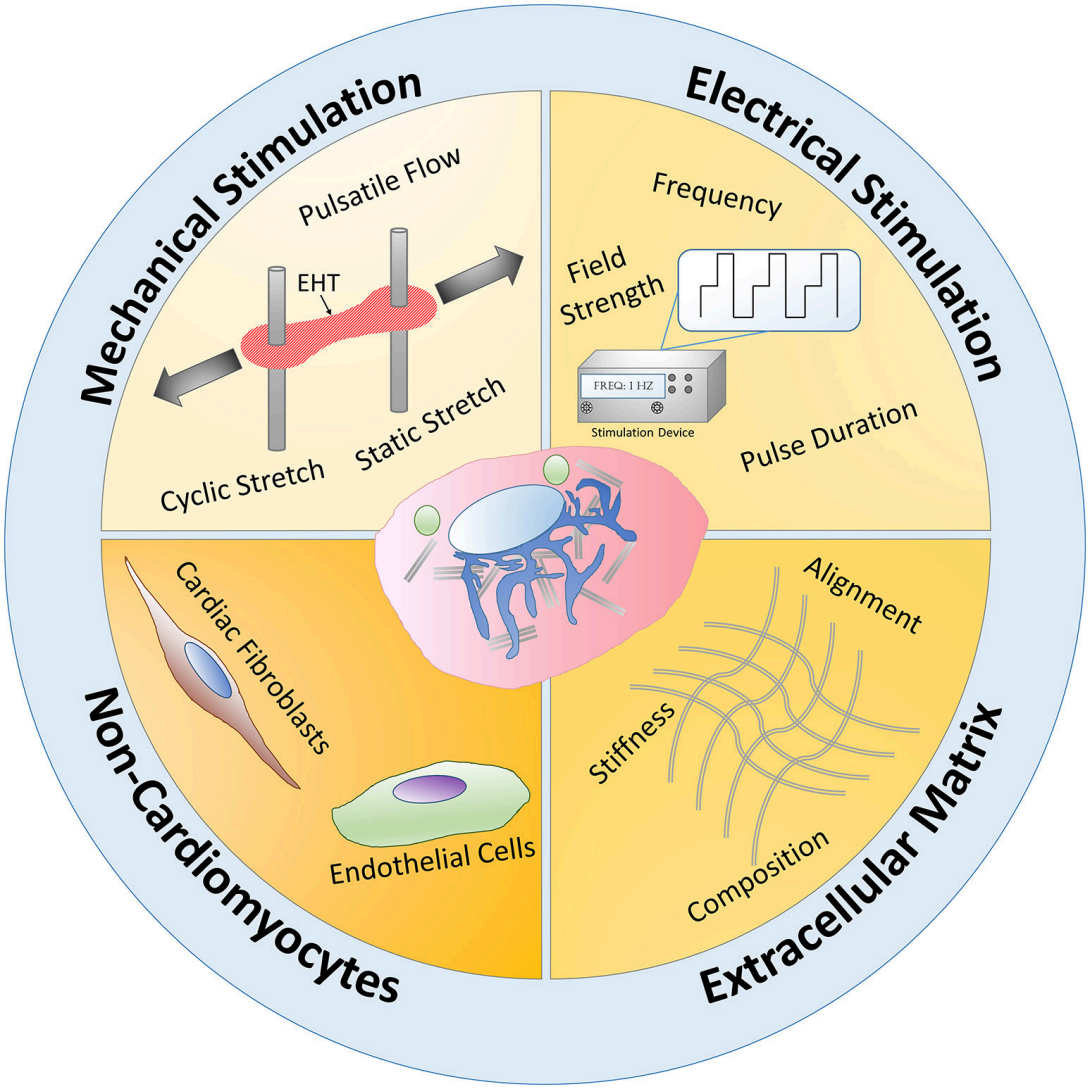
**Natural engineering approaches schematic**. Mechanical stimulation, electrical stimulation, extracellular matrix interactions, and non-cardiomyocyte interactions have been utilized to mimic the natural physiological conditions of CMs during development, known as natural engineering, in order to improve PSC-CM maturity. Mechanical stimulation in the form of pulsatile flow, static and cyclic stretch mechanisms have increased engineered heart tissue (EHT) maturity. Applying an electrical stimulus to PSC-CMs and modulating the frequency, pulse duration, and field strength has led to some functional and structural PSC-CM maturation improvements. By using natural inspired extracellular matrix interactions, such as alignment, ECM composition, and stiffness changes, researchers have been able to increase PSC-CM maturity. Non-cardiomyocyte interactions with PSC-CMs have also led to increased maturation. Overall, these four main natural engineering approaches occur simultaneously and synergistically during the natural developmental paradigm *in vivo* and therefore need to be recapitulated *in vitro* to further improve PSC-CM maturity.

Several promising advances of cardiomyocyte maturation have been made through the utilization of *in vitro* mechanical stimulation, electrical stimulation, extracellular matrix interactions, and co-cultures with non-cardiomyocytes, which mimic the natural physiological environment of CMs *in vivo*, known as natural engineering (Figure 5). Nonetheless, even with these natural engineering approaches, human PSC-CMs have yet to surpass the natural fetal stages of CM development (Feric and Radisic, 2016). The majority of *in vitro* maturation studies only assess a few maturation parameters and thus do not capture the full picture of where their PSC-CMs lie within the natural stages of CM development. These studies also usually choose random undefined ending time points and thus most likely are not providing enough time for their CMs to reach their maximum maturity within their systems. Longer culture times have proven to help with overall PSC-CM maturity, even in 2D culture (Kamakura et al., 2013). Most studies similarly only compare PSC-CM maturity to either 2D culture or unstimulated controls. Future studies should strategically use various developmentally staged native CMs within their naturally engineered systems as controls for direct comparison to their PSC-CMs. By utilizing developmentally staged native CMs, researchers will be able to more accurately determine how well their systems can improve CM maturity according to the natural CM developmental stages and also pinpoint the gaps in maturity between their PSC-CMs and native CMs within their specific systems. The following sections discuss the variety of natural engineering approaches researchers have used in an attempt to improve CM maturity *in vitro*.

### Maturation of cardiomyocytes by natural developmentally inspired stimulation strategies

The importance of *in vitro* mechanical and electrical stimulation on CM maturation have been known for over a decade (Zimmermann et al., 2000; Radisic et al., 2004). Many studies have developed bioreactor systems that subject CMs seeded in a three-dimensional matrix to create engineered heart tissue (EHT) to these stimuli. However, many systems do not employ a natural engineering program since they use uninspired, non-physiological values. Therefore, this review discusses some recent approaches that are natural developmentally inspired to improve overall CM maturation. Table 1 summarizes this information. Some recent thorough reviews on mechanical and electrical stimulation of CMs can be found elsewhere (Rangarajan et al., 2014; Cao et al., 2015; Stoppel et al., 2016).

**Table 1:** **Stimulation strategies for improving cardiomyocyte maturity**.

**Stimulation type**	**Stimulation device**	**Stimulation regimen**	**Cardiac construct**	**Maturation parameters**	**Advantages**	**Limitations**	**References**
**Fluid flow, pressure, and strain**	Microfluidic cell culture system with combined cyclic fluid flow, chamber pressure, and strain	• Duration: 4 days • 10 mmHg and 8-15% cyclic stretch at 2 Hz • Fluid flow rate of 44μL/cycle	2D culture of embryonic chick CMs on flexible collagen matrix	• Increase in contractility, cardiac troponin T, and SERCA	• Multiple physiologically relevant types of stimulation	• 2D cardiac constructs	Nguyen et al., 2015
	Biomimetic pressure gradient mechanical stimulation system for mimicking cardiac cycle with fluid flow	• Duration: 3 days • 50 mmHg pressure • 10% biaxial stretch • Gradual vs immediate physiological strain	HIPS-CMs on collagen/matrigel coated flexible PDMS membrane	• N/A	• Mimics physiological cardiac cycle • Cell culture chamber performs pumping action to move fluid	• Cells cultured on surface of 3D gels	Rogers et al., 2016
**Cyclic strain**	Adapted the commercialized Flexcell apparatus for cyclic uniaxial stretch by use of nylon mesh anchorage points	• Duration: 7 days • 5% cyclic stretch at 1 Hz	HPSC-CMs within collagen/Geltrex gel anchored to nylon mesh	• Increase in beta-myosin heavy chain, cardiac troponin, L-type calcium channel and ryanodine receptor expression • Increase in sarcomere alignment and Z-disk formation	• Uniaxial aligned constructs • Co-culture with endothelial cells	• Anchored nylon mesh ends can cause some stress concentrations and necrosis	Tulloch et al., 2011
	Uniaxial mechanical stimulation device driven by electromagnetic force that actuates two stainless steel tissue clamps	• Duration: 3 days • 12% cyclic uniaxial stretch at 1 Hz	HESC-CMs in gelatin sponges (Gelfoam)	• Increase in cellular size and alignment • Increase in Z disk organization • Increase in KCNJ2 expression • Increase in electrical coupling • Increased integration with host tissue *in vivo*	• Non-contact actuation through use of electromagnetic force	• Clamps cause major stress concentrations and tissue necrosis	Mihic et al., 2014
	Cyclic uniaxial stretch using linear motor that moves tissue clamp with perfusable vascular matrix	• Duration: 2 days • Flow rate: 0.2 mL/min • 5% cyclic uniaxial stretch at 1 Hz	Decellularized porcine submucosa, decellularized porcine vascular matrix, and neonatal rat CMs in collagen/matrigel	• Increase in cardiac troponin T, connexin-43 and myosin heavy chain expression	• Cyclic stretch combined with perfusable vascular network • Large cardiac patch	• Clamps can cause stress concentrations and necrosis	Lux et al., 2016
**Electrical stimulation**	Cardiac biowire with electrical stimulation by electrodes and perfusion through wire	• Duration: 7 days • 0-3 Hz and 0-6 Hz • Perfusion rate: 2 μL/min	HPSC-CMs in collagen/ matrigel compacted around perfusable poly-tetrafluoroethylene wire suture	• Increase in connexin-43 and cardiac troponin • Increase sarcomeric maturity (Z disks, H zones, and I bands)	• Mimics myocardial fibers • Two types of stimulation • Functional perfusion system can be used to test drug response	• Limited perfusion permeability (does not allow protein transport) • No mechanical actuation	Nunes et al., 2013a; Xiao et al., 2014
**Electrical stimulation and cyclic strain**	Electrical stimulation by electrodes and mechanical stimulation by pneumatic inflation of tube	• Duration: 14 days • 5% cyclic strain at 1 Hz • 3 V/cm, 1 Hz, and 1 ms duration for electrical stimulation • Simultaneous and delayed regimens	Neonatal CMs in fibrin hydrogel ring	• Increase in cardiac troponin and SERCA • Increase in Akt protein expression (involved in hypertrophy)	• Contiguous tissue constructs that prevent stress concentrations and necrosis • Combined electrical and mechanical stimulation • Control over timing of each stimulation type	• Tissue ring constructs not representative of physiological geometry	Morgan and Black, 2014
**Electrical stimulation and static stress**	Electrical stimulation and adapted commercialized Flexcell apparatus for static uniaxial stress by use of nylon mesh anchorage points	• Duration: 2 weeks • Static stress • 5V/cm, 2 Hz, and 5 ms pulses for electrical stimulation	HIPS-CMs within collagen gel anchored to nylon mesh (same system as Tulloch et al.)	• Increase in SERCA and RYR expression • Increase in cellular alignment and size • Increase in contractility and electrical coupling	• Uniaxial aligned constructs • Combination of static stress and electrical stimulation	• Anchored nylon mesh ends can cause some stress concentrations and necrosis	Ruan et al., 2016
	Point electrical stimulation through embedded platinum wires and static stress by biofabricated microtissues anchored around PDMS posts	• Duration: 7 days (3 days without electrical stimulation) • Static stress • 6 V/cm, 1 Hz, and 1 ms pulses for electrical stimulation	HESC-CMs within collagen gel compacted around two PDMS posts to create cardiac microtissues	• Increase in sarcomere alignment/ organization • Increase in myosin light and heavy chains • Increase in conduction velocity • Increase in cellular alignment	• Point electrical stimulation • Contiguous uniaxial aligned constructs • Co-culture with fibroblasts • Combination of static stress and electrical stimulation	• No mechanical actuation	Thavandiran et al., 2013

#### *In vitro* mechanical stimulation

One specific natural engineering approach involves the use of mechanical stimulation bioreactor systems within *in vitro* culture to recapitulate physiological *in vivo* mechanical stimuli, such as cyclic stretch and pulsatile flow. One specific study involved culturing CMs from embryonic chick hearts on a novel custom microfluidic cell culture system that allowed three different forms of mechanical stimulation: (1) cyclic fluid flow, (2) chamber pressure, and (3) strain, to recapitulate the various mechanical stimuli *in vivo*. Compared to static controls, the mechanically stimulated embryonic CMs had an increased expression of cardiac troponin and SERCA, as well as an increase in contractility (Nguyen et al., 2015). However, a significant drop of cardiac specific genes occurred when chick embryonic CMs were isolated and cultured *in vitro* compared to the CMs that remained within the native ventricular tissue (Nguyen et al., 2015). This data indicates the large gap between *in vitro* natural engineering and the *in vivo* natural developmental program.

**Figure 6:**
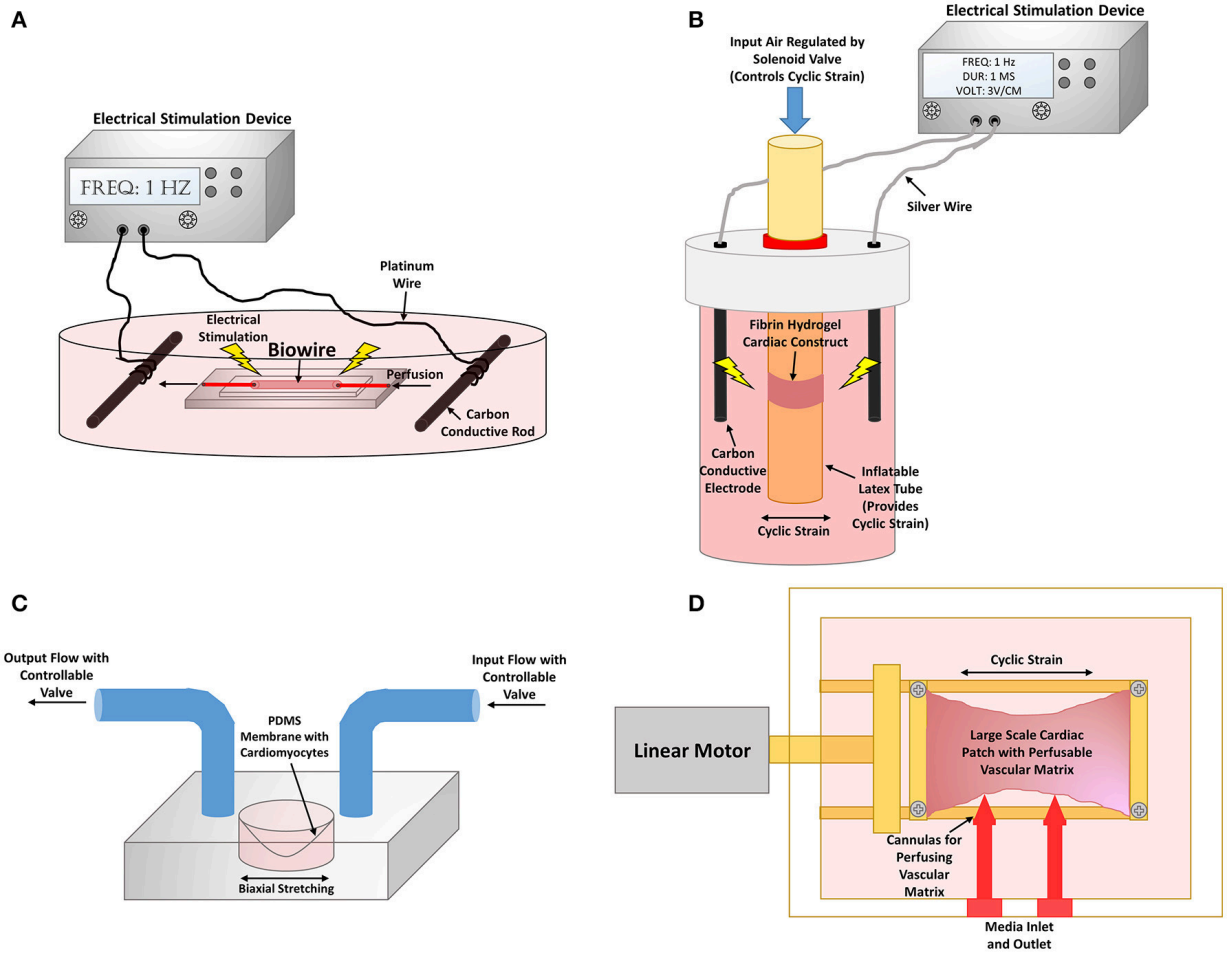
**Bioreactor stimulation systems to improve cardiomyocyte maturity. (A)** Cardiac biowire system where PSC-CMs are cultured on a perfusable wire to mimic myocardial fibers. The cardiac biowires are also equipped to be electrically stimulated through two carbon electrodes connected to an electrical stimulation device. Schematic representation of bioreactor from Xiao et al. (2014). **(B)** A combined mechanical and electrical stimulation bioreactor system. Fibrin hydrogel cardiac constructs are cultured on an inflatable latex tube that can provide cyclic strain to the tissue constructs via a pneumatic system. Exogenous electrical stimulation can also be applied to the constructs by the use of two carbon electrodes connected to an electrical stimulation device. Schematic representation of bioreactor from Morgan and Black (2014). **(C)** A biomimetic system where PSC-CMs are seeded on to a flexible membrane that can be strained to perform the pumping action required to move fluid. Pressure gradients are used within the input and output to recapitulate each aspect of the cardiac contraction cycle. Schematic representation of bioreactor from Rogers et al. (2016). **(D)** Large cardiac patch that is cyclic strained by the use of a linear motor that actuates one of the tissue clamped ends. Cannulas are also inserted into the vascular matrix for perfusion of the construct. Schematic representation of bioreactor from Lux et al. (2016).

Another interesting approach involved a bioreactor system that combined cyclic stretch and pulsatile flow of a large implantable EHT. The cardiac patch was composed of decellularized porcine small submucosa, a decellularized porcine vascular matrix seeded with endothelial cells, and neonatal rat CMs seeded within a collagen and matrigel scaffold (Lux et al., 2016). Although not explicitly stated in many papers, nutrient diffusion is a major limiting factor for large static EHTs since diffusion of oxygen can only occur through about a 100 μm thickness. Consequently, the interior of the constructs often contain apoptotic cells which limit the maturity, without the use of perfusion to facilitate nutrient delivery (Gerecht-Nir et al., 2003). This custom bioreactor cyclically stretched the large EHT at 5% stretch and 1 Hz, and perfused the vascular matrix with a flow rate of 0.2 mL/min (Figure 6D). This dual perfusion and cyclically stretched EHT led to an increase in several maturation parameters, such as cardiac troponin, connexin-43, and MHC expression. The endothelial network within the constructs were more complexly formed in the stimulated EHTs (Lux et al., 2016). This study was one of the first to combine endothelial cells, CMs, a perfusable vascular network, along with cyclic stretch and perfusion to create EHTs. These combined naturally engineering approaches are necessary to obtain more physiologically-relevant EHTs that mimic natural myocardial tissue.

PSC-CM maturation via mechanical stimulation has not been studied quite as readily as other CMs. One particular study involved culturing EHT composed of human PSC-CMs within a collagen/Geltrex gel suspended on the commercialized Flexcell apparatus, which had a silicon bottom and anchors of nylon mesh. The apparatus cyclically strained the EHTs at 5% elongation and 1 Hz, a physiologically relevant frequency. Within 4 days, there was an upregulation of several key cardiac genes, such as β-MHC, cardiac troponin, L-type calcium channels, and RyR. At day five of culturing, the human PSC-CMs had increased cellular size and alignment as well as synchronous beating. Within 7 days, sarcomeric structural organization greatly improved with aligned and distinguishable Z disks. When the EHTs were co-cultured with human umbilical vein endothelial cells, vessel structures began to form as well. This small vasculature partly anastomosed with the host vasculature when implanted within an injured rat heart, which provided evidence of the benefits of co-culturing with non-CMs (Tulloch et al., 2011).

Mechanically stimulated EHTs after implantation on *in vivo* rat ischemic hearts have also been assessed. Human ESC-CMs were cultured in a gelatin sponge and underwent uniaxial cyclic stretching at 12% elongation at 1 Hz for a total of 3 days. The *in vitro* results showed an increase in cellular size, augmented connexin-43 expression, more organized Z disks, upregulation of several ion channels including KCNJ2, and also more efficient electrical coupling. When implanted *in vivo*, the mechanically stimulated EHTs showed greater unity with the host tissue and less myocardial wall thinning compared to the static control and acellular control (Mihic et al., 2014). This study supports the need for *in vitro* manipulation of CM maturation prior to transplantation *in vivo*.

Rogers et al. developed a biomimetic system that emulates the physiological cardiac cycle of ventricular stretching, contraction, ejection, and relaxation (Figure 6C). This system design contained human IPS-CMs seeded on a collagen/matrigel coated flexible PDMS membrane that provided the pumping action by the use of a pressure gradient to move fluid through the system, and therefore is more physiologically relevant than using a pump to perform the hemodynamic flow. The study assessed gradual vs. immediate application of physiological adult ventricular mechanical loads of 50 mmHg systolic pressure and 10% biaxial stretching. Although the study did not necessarily determine any significant differences in IPS-CM maturation between stimulated conditions and static controls, the results provided evidence that gradual application of physiological loading is necessary in order to increase cell survivability (Rogers et al., 2016).

#### *In vitro* electrical stimulation

To mimic the electrophysiological development of CMs *in vivo*, researchers have utilized exogenous electrical stimulation in hopes of maturing CMs to a more adult-like phenotype. These exogenous electrical stimulation bioreactor designs usually involve two electrodes composed of erosion resistant materials that direct electrical current through the CMs.

One system utilized a “cardiac biowire” system to promote alignment and thus mimic natural CM anisotropy found *in vivo*. Collagen/matrigel seeded with human PSC-CMs compacted around an anchored sutures were electrically stimulated gradually from 0 to 3 Hz for 7 days, since 3 Hz is equivalent to the average human fetal heart rate of about 180 bpm, and tested 0–6 Hz for 7 days to determine the limits of the EHTs. Surprisingly, the aligned EHTs that were stimulated using the gradual 0–6 Hz had increased cellular surface area, and sarcomere organization with H zones, I bands, and Z disks present, and more intercalated discs and mitochondria formed. The system improved the electrophysiological function with an increase in conduction velocity, KCNJ2 expression, and synchronous beating. The authors hypothesized that the non-physiological high gradual frequency had greater improvements in maturity since other factors important to cardiac development were not implemented in the system, such as non-cardiomyocytes (Nunes et al., 2013a).

PSC-CMs also mature when electrically stimulated. One study involved human ESC-CMs and human ESC-derived cardiac fibroblasts in a collagen/matrigel scaffolds cultured in either a uniaxially aligned contiguous construct around two PDMS posts or biaxially aligned contiguous construct around eight PDMS posts. Unlike other electrical stimulation designs, this system provided point stimulation by implanted platinum wires within the microwells to allow for activation of the cells in sequence, which is more physiologically-relevant compared to whole field stimulation with two electrodes. The results show that the uniaxial constructs co-cultured with fibroblasts had greatest improvements of maturity with highly aligned sarcomeres and upregulation of MLC isoforms (Thavandiran et al., 2013).

#### Combination of *in vitro* electrical and mechanical stimulation

Researchers have developed bioreactors that combine both stimuli to further maximize CM maturity. Several non-PSC-CMs have been used in combined bioreactor studies. One significant novel approach involved a combined bioreactor system that analyzed the importance of when to administer the mechanical and electrical stimulations. Five test conditions were studied: static, mechanical stimulation only, electrical stimulation only, synchronized electrical and mechanical stimulation, and delayed electrical and mechanical stimulation which was meant to mimic physiological isovolumetric contractions. The EHTs, which were composed of neonatal CMs seeded in a tubular fibrin hydrogel construct, were subjected to 5% cyclic strain and electrical stimulation of 3 V/cm, 1 Hz, and 1 ms durations (Figure 6B). The results showed that the isovolumetric contraction condition led to greater expression of SERCA and cardiac troponin as well as increasing amounts of AKT, which is involved in physiological hypertrophy (Morgan and Black, 2014).

PSC-CMs subjected to combined stimuli have also shown to increase maturity. One approach involved improving the cardiac biowire system that was previously described by including perfusion through the polytetrafluoroethylene wire suture (Figure 6A). This adjustment led to an increase in connexin-43 and cardiac troponin expression for the seeded PSC-CMs (Xiao et al., 2014). Ruan et al. seeded hIPS-CMs within collagen matrix subjected to electrical stimulation of 5 V/cm, 5 ms duration, and 2 Hz as well as static stretch. The combined stimulation led to significant improvements in contraction force which reached 1.34 ± 0.19 mN/mm^2^ as well as RYR and SERCA expression compared to either treatment condition alone (Ruan et al., 2016). These studies further indicate the importance of synergistically combining natural engineering approaches to more closely resemble the natural developmental program.

#### Current limitations of *in vitro* stimulation strategies

Although progress has been made in the maturation of CMs by the use of mechanical and electrical stimulation, several limitations still remain. Varying the regimen can lead to major changes in CM maturation since variability is a natural part of cardiac function (Musialik-Łydka et al., 2003; Stoppel et al., 2016). By testing more regimens that are inspired from the natural developmental program, researchers will be able to obtain a deeper understanding of the optimal mechanical stimulation regimens that induce the greatest amount of PSC-CM maturity. Depending on the mechanical or electrical stimuli, physiological or pathological hypertrophy can be activated, providing evidence for the importance of optimizing the regimens (Bernardo et al., 2010). Confounding variables also are associated with comparing one stimulation study to another. Each study uses different differentiation protocols to obtain PSC-CMs and therefore the maturity and purity of the PSC-CMs varies even before any stimulation is applied (Talkhabi et al., 2016). The compositions of the biomaterials used for the scaffolds differ between studies, which has an effect on CM maturity and other cellular processes (Gershlak et al., 2013). One major challenge of EHT electrical stimulation is the fact that most biomaterials used for the scaffold have a poor electrical conductivity (You et al., 2011; Lee et al., 2014). The composition, thickness, and overall geometry of the cell environment play a large role in the electrical conductivity (Sedmera et al., 2004; Sankova et al., 2012). The CMs are most likely experiencing heterogeneous electrical and mechanical stimuli within the scaffolds and thus various induced maturity.

### Maturation of cardiomyocytes by natural developmentally inspired extracellular matrix

The studies that were discussed in the electrical and mechanical stimulation sections utilized more basic ECM compositions for their scaffolds. Although these ECM components provided a 3D microenvironment for the CMs, the ECM of these EHTs do not effectively recapitulate many aspects of the natural cardiac ECM that are known to drive the natural developmental program of CMs. As shown in studies that modulated the topographical alignment, stiffness, and/or the overall material composition of the scaffolds being used, the ECM actively influences CM maturation *in vitro*. Table 2 summarizes this section.

**Table 2:** **Naturally engineered extracellular matrix scaffold strategies for improving cardiomyocyte maturity**.

**Types of ECM**	**ECM scaffold design**	**Cell source**	**Results**	**References**
**Engineered biological**	Collagen coated thiolated-hyaluronic acid hydrogels with increasing stiffness by using greater crosslinking weights	Embryonic chick cardiomyocytes	• Increase in NKX2.5 and cardiac troponin • Increase in sarcomere organization	Young and Engler, 2011
	Collagen/matrigel scaffold in recapitulated anisotropic orientation of anisotropic heart fibers anchored around PDMS posts	Neonatal rat cardiomyocytes	• Transverse tubule formation • Increase in sarcomere organization, conduction velocity and calcium kinetics	Bian et al., 2014a
**Adult cardiac extracellular matrix**	Decellularized adult zebrafish ventricle	N/A (implanted as ECM-only *in vivo* on infarcted murine hearts)	• Decreased left ventricle dilation • No increase in progression of inflammation or fibrosis • Increase in heart's overall contractility • Increase in cardiomyocyte proliferation	Chen et al., 2016
	Decellularized whole murine adult hearts	HIPS-CMs, hIPS-endothelial cells, hIPS-smooth muscle cells	• Increase in cardiomyocyte proliferation throughout scaffold • Increase in myofilament size • Increase in cardiac troponin, myosin heavy chain, and connexin-43	Lu et al., 2013
	Laser cut decellularized adult porcine ventricular myocardium	PSC-CM	• Increase in contraction strength and sarcomere organization • Increase in connexin-43, N-cadherin, cardiac troponin	Schwan et al., 2016
	Decellularized adult human cadaver whole hearts	HIPS-CMs	• Increase in cardiac troponin and myosin heavy chain • Increase in sarcomere organization	Guyette et al., 2016
**Embryonic cardiac extracellular matrix**	Decellularized embryonic day 18.5 mouse hearts	Mouse ESC-derived cardiac progenitors	• Differentiation into CMs as evidence by spontaneous beating • Increase in alpha-actinin	Chamberland et al., 2014
	Decellularized embryonic day 18 mouse hearts and adult mouse hearts	Cardiac progenitor cell line	• Increase in cell proliferation in fetal hearts • No cardiac troponin expression	Silva et al., 2016
	Decellularized bovine fetal and adult hearts solubilized into gel	HIPS-CM	• Increase in calcium handling and ion channel genes within 3D culture • Greatest increase in maturation markers in solubilized decellularized adult bovine gel	Fong et al., 2016

#### Examples of stiffness to promote maturity

Studies have been performed on assessing CM maturity in response to material stiffness. Researchers have developed tunable hydrogels to mimic the changes in stiffness that occur during development. Embryonic chick CMs were cultured on collagen coated thiolated-hyaluronic acid hydrogels. The stiffness of the material was increased during the culture by using higher crosslinking weights for 2 weeks. The CMs had significant increases in several cardiac specific genes, such as NKX2.5 and cardiac troponin, as well as more mature sarcomeric structures compared to less stiff conditions (Young and Engler, 2011). This group also assessed which mechanosensitive pathways are involved in this maturation and determined that AKT and p38 MAPK are significantly upregulated within embryonic CMs that undergo dynamic increases in stiffness (Young et al., 2014).

#### Examples of extracellular matrix composition to promote maturity

Although stiffness can change CM maturity, the ECM composition affects the cells ability to sense the different mechanical forces. In one study, decellularized ECM from fetal, neonatal, and adult rat hearts were solubilized within PA gels of varying stiffness and seeded with mesenchymal stem cells. As the stiffness increased, a decrease in traction stress was observed for adult ECM PA gels. However, the exact opposite occurred on fetal ECM PA gels (Gershlak et al., 2013). Therefore, ECM composition is an important factor in influencing cellular behavior.

##### Decellularized cardiac ECM

One promising technique is the use of decellularized cardiac ECM (dcECM) from a variety of different animal models to promote CM maturity. DcECMs have several advantages: (1) contain anisotropic alignment of native heart, (2) contain essential cardiac ECM components, and (3) contain a vasculature template that can promote vasculature development. With these advantages comes some drawbacks as well. Preserving the entire native ECM has proven to be difficult since decellularization protocols end up disrupting portions of the ECM network while trying to eliminate all cellular components. There is a fine balance between proper decellularization and preservation of the native ECM. Most decellularization protocols involve a cell lysis step, followed by a cell removal detergent step, and then a proteolysis and DNAse/RNAse step to eliminate any remaining cellular components. Convection through the use of agitation shakers or perfusion apparatuses are usually used in combination to allow the reagents to effectively penetrate the tissue (Moroni and Mirabella, 2014).

DcECMs are highly bioactive and thus can be used even without reseeding cells to partially promote cardiac regeneration within *in vivo* infarct heart models. One study on a rat model with chronic myocardial infarction was repaired with an adult ventricular porcine dcECM that showed recruitment of host cardiac progenitors and CMs into the scaffold, vascularization penetrating the scaffold, as well as some positive remodeling responses (Sarig et al., 2016). Adult ventricular zebrafish dcECM also holds promising potential as a scaffold for mammalian cardiac regeneration. Zebrafish can regenerate their heart and thus one particular study wanted to take advantage of this capability. When this zebrafish dcECM was implanted within acute infarcted murine hearts, functional improvements occurred. There was no increase in fibrosis or inflammation, the dilation of the left ventricle was lessened, the heart contractility increased, and CM proliferation increased within the infarcted region. Neuregulin-1, which is known to increase survival and growth of CMs, was shown to be present within the zebrafish dcECM and thus was involved in the regenerative capabilities (Chen et al., 2016). These studies indicate the promising potential associated with dcECM as a scaffold for cardiac regeneration.

DcECMs have also been reseeded with CMs *in vitro* to assess their potential as a scaffold. Lu et al. decellularized whole murine hearts and reseeded with human IPS-derived cardiovascular progenitor cells that were then differentiated into CMs, smooth muscle cells, and endothelial cells. The proliferation of the CMs doubled within the scaffold, with an increase in expression of connexin-43, both MHC isoforms, and cardiac troponin. The action potentials were still irregular however (Lu et al., 2013). Schwan et al. laser cut cardiac muscle strips from porcine ventricular myocardium, decellularized the ECM, mounted the dcECM on a special clamp system, and reseeded the dcECM with PSC-CMs. The use of this aligned dcECM as an EHT scaffold improved the contraction strength and sarcomere organization, and augmented connexin-43, N-cadherin, and cardiac troponin expression (Schwan et al., 2016).

**Figure 7:**
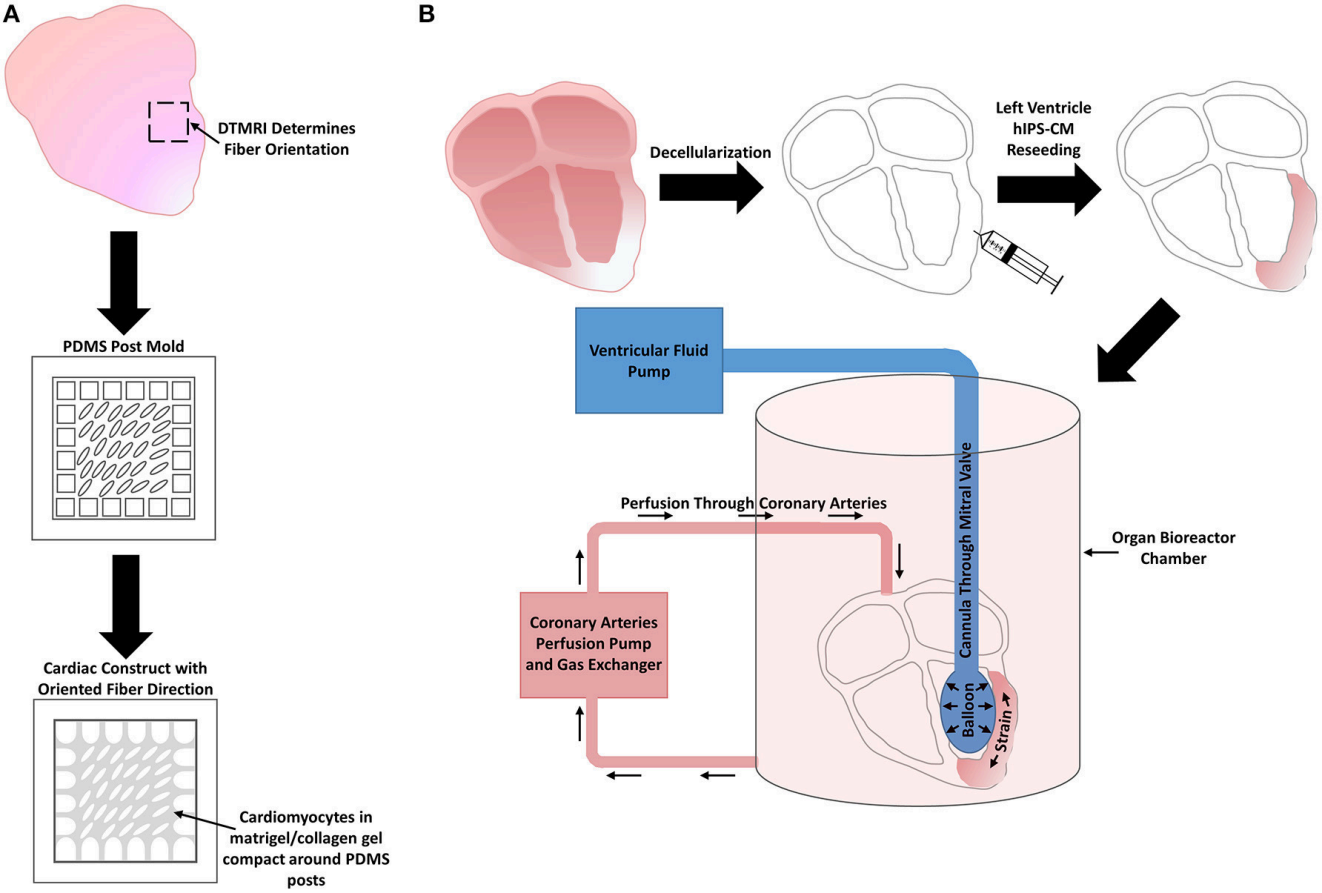
**Extracellular matrix scaffold designs. (A)** Diffusion tensor magnetic resonance imaging (DTMRI) was used to determine the anisotropic fiber orientation of the heart wall. This information was then translated into a PDMS mold that represent that correct fiber orientation. Cardiomyocytes in a matrigel/collagen gel are seeded and allowed to compact around the PDMS posts to create a cardiac construct that recapitulates the anisotropic orientation of the myocardial fibers. Schematic representation of construct design from Bian et al. (2014a). **(B)** Whole adult human cadaver hearts are decellularized and the left ventricular myocardial wall is reseeded with human IPS-CMs. The reseeded hearts are then placed in an organ chamber bioreactor system that pumps fluid into a balloon that is inserted into the left ventricle to strain the seeded human IPS-CMs. A perfusion system is also attached to the coronary arteries to ensure proper nutrient exchange occurs. Schematic representation of construct design from Guyette et al. (2016).

Another novel technique involves decellularizing adult whole hearts. One early study reseeded rat CMs on decellularized rat hearts that were connected to a bioreactor that applied mechanical stimulation by perfusion and electrical stimulation at the apex (Ott et al., 2008). Building off this earlier study to create a more clinically relevant model, decellularized cadaveric human adult hearts were reseeded with human IPS-CMs and placed within a bioreactor system that provided perfusion through the coronary arteries as well as left ventricular wall strain by use of a modulatory pressure balloon for 14 days (Figure 7B). The seeded human IPS-CMs were able to contract readily, were metabolically active, had high cell viability, and expressed various cardiomyocyte markers, such as MHC and cardiac troponin T. Various ranges of IPS-CM maturity were observed. Some cells had disorganized sarcomeres while others containing more organized sarcomeres with some striations but the overall sarcomere maturity remained within the fetal CM developmental stages (Guyette et al., 2016).

Besides adult ECM, fetal ECM offers developmental signals that can drive beneficial cardiomyocyte responses and thus potentially can be used as an advantageous scaffold for cardiac regeneration. Fetal ECM provides more proliferation signals for CMs as shown in a study comparing decellularized rat fetal (day 18–19), neonatal, and adult hearts seeded with neonatal CMs (Williams et al., 2014). Chamberland et al. decellularized embryonic murine hearts at day 18.5 of development and reseeded with mouse ESC-derived cardiac progenitor cells. The fetal dcECM allowed for proper proliferation throughout the entire scaffold and spontaneously beating after 1 day indicating some of the cells differentiated into CMs. Alpha-actinin expression also was shown to be augmented (Chamberland et al., 2014). Another study involved decellularizing fetal murine hearts at day 18 of development and adult murine hearts. After reseeding with a cardiac progenitor cell line, the fetal dcECM showed greater cellular proliferation than the adult dcECM but no cardiac troponin was present in either scaffold. Higher levels of several factors involved in CM proliferation and improving cardiac repair, such as transforming growth factor-beta, periostin, fibronectin, and heparin-binding epidermal growth factor were present within the fetal dcECMs (Silva et al., 2016). Decellularized fetal (developmental day was not reported) and adult bovine myocardium's potential to improve hIPS-CM maturity was compared in another study. The dcECMs were digested and implemented into a non-reported hydrogel material before seeding. The results show that significantly higher expression levels of key maturation genes occurred with human IPS-CMs seeded on adult dcECMs (Fong et al., 2016). Digesting the dcECM completely disrupts the organization of the ECM and thus may have played a role in the differences in maturity that were seen when comparing fetal and adult dcECM seeded with human IPS-CMs.

Although these studies show mixed results in terms of the potential for fetal dcECM as an advantageous scaffold for cardiac regeneration, the fetal hearts chosen for these studies were already completely developed. Rat hearts and mouse hearts at embryonic day 18 have finished cardiogenesis (Savolainen et al., 2009; Marcela et al., 2012). These late developmental fetal dcECMs may not provide the optimal developmental signals to drive beneficial PSC-CM cellular responses, such as proliferation, differentiation, and maturation. *In vivo*, the cardiac ECM changes rapidly during cardiogenesis, with major alterations in the composition, organization, and stiffness, which have shown to modulate CM behavior (Gershlak et al., 2013; Takahashi et al., 2013). Therefore, future studies should take advantages of these natural changes in cardiac ECM by investigating the effect of various developmentally staged fetal dcECMs on PSC-CM maturity.

##### Bioprinting ECM

Bioprinting is another promising technique since one can control the patterning of the constructs with a precise density and distribution of cellular and ECM components to mimic the myocardial structure (Borovjagin et al., 2017). A recent study created EHTs using an alternating bioprinted pattern of two bioinks: solubilized porcine dcECM seeded with human cardiac progenitor cells, and solubilized porcine dcECM seeded with mesenchymal stem cells and vascular endothelial growth factor for a pre-vascularized network. Results showed an increase in *in vitro* CM maturity by upregulation of cardiac troponin and alpha-actin, and improved cardiac function after implantation within infarcted rat hearts with increased graft vasculature (Jang et al., 2017).

#### Examples of engineered topographical alignment to promote maturity

Overall alignment of CMs is involved in the effectiveness of contractions and electromechanical coupling of cardiomyocytes. The anisotropy allows for action potentials to propagate in a specific direction and thus greatly improves the electrical conduction of CMs (Bian et al., 2014b). Due to these facts, researchers have developed ways to promote CM alignment, and thus maturity, through various engineered topographical cues. Studies have developed micropatterned grooves that enhanced cellular alignment and resulting CM maturity of 2D culture systems (Salick et al., 2014; Carson et al., 2016). Engineered nanofibrous scaffolds have been produced that mimic the natural fibrous cardiac ECM (Khan et al., 2015; Rath et al., 2016). 3D constructs have been created to promote CM alignment, such as the biowire technology (Nunes et al., 2013a), and aligned and contiguous EHTs around flexible posts (Zhang et al., 2013; Hirt et al., 2014).

Another novel natural engineering approach involved using diffusion tensor magnetic resonance imaging to determine the fiber directions within the native heart wall (Figure 7A). Using this information, rectangular EHTs composed of neonatal CMs and collagen/matrigel were developed that recapitulated the anisotropic heart fibers. Results showed highly aligned CMs, developed transverse tubules, increased calcium kinetics, increased conduction velocities, and greater sarcomere organization (Bian et al., 2014a). Resembling physiological fiber orientation increases EHT functional maturity toward a more clinically relevant model.

### Maturation of cardiomyocytes by natural developmentally inspired culture with non-cardiomyocytes

Due to the essential role of non-cardiomyocytes during normal physiological development and function of the heart wall, various researchers have investigated the use of non-cardiomyocytes within *in vitro* co-cultures to drive cardiomyocyte maturation. To create a more robust, physiologically-relevant cardiac construct, other cell types are necessary, such as cardiac fibroblasts and endothelial cells. As a result, researchers are moving away from CM-only EHTs and are beginning to investigate cardiomyocytes with other cell types.

Many studies with neonatal or fetal CMs co-cultured with fibroblasts and/or endothelial cells functionally improved and matured the CMs (Narmoneva et al., 2004; Radisic et al., 2008; Garzoni et al., 2009; Iyer et al., 2012; Vuorenpää et al., 2014). One particular interesting study involved co-culturing embryonic quail EPDCs with neonatal mouse CMs *in vitro*. When the EPDCs were directly connected to the CMs, the proliferation, connexin-43 expression, N-cadherin expression, cellular alignment, and contractility all significantly increased for CMs (Weeke-Klimp et al., 2010).

Kim et al. was one of the first groups to conduct co-culture experiments of non-cardiomyocytes with human PSC-CMs. This group cultured human ESC-CM spheroids with smaller clusters of embryonic non-cardiomyocyte cells, which contained the majority of the heart cell types. These interactions led to functional improvements in ion channel profiles and electrophysiology with an increase in the action potential amplitude, and more negative resting membrane potential (Kim et al., 2009). Another co-culture system involved the use of an endodermal cell line directly connected to mouse and human pluripotent stem cells, which led to the differentiation of the cells into cardiac progenitors that expressed the cardiac specific genes of NKX2.5 and Isl1 (Uosaki et al., 2012).

Several studies have cultured PSC-CMs with fibroblast cells. Thavandiran et al. generated EHTs composed of human ESC-CMs and human ESC-CFs in a collagen/matrigel construct that were also electrically stimulated. They demonstrated that the optimal CF to CM ratio was 3:1, which most significantly increased the CM maturity. This optimized co-culture ratio resulted in increases in cardiac specific genes, such as MHC and MLC isoforms, increased conduction velocity, enhanced overall tissue integrity, greater sarcomere organization, better cellular alignment, and promotion of beneficial collagen matrix remodeling (Thavandiran et al., 2013). Improvements in differentiation efficiency can be achieved by CM co-cultures. One experiment involved mouse ESCs in a collagen gel indirectly co-cultured with CFs that were placed into a well insert that sat above the mouse ESCs. This 3D indirect co-culture system led to greater CM differentiation efficiency and improved sarcomere organization, and connexin-43, alpha-actinin, and cardiac troponin expression (Ou et al., 2011).

The combination of more than one cell type with PSC-CMs further improves the EHTs maturity. Human IPS cells were differentiated into endothelial cells, cardiomyocytes, and vascular smooth muscle progenitors and seeded within a collagen/matrigel 3D construct to assess the multi-culture systems functional maturity. The combination of all three cell types improved the EHT's contractile force, cellular alignment, and sarcomere organization *in vitro*. When implanted *in vivo* on infarcted rat hearts, vasculature was present within the EHTs that originated from the host as well as the construct itself and some functional heart improvements occurred (Masumoto et al., 2016). Roberts et al. engineered large scale vascularized EHTs containing human ESC-CMs co-cultured with human dermal fibroblasts in a high density collagen matrix that contained an engineered microvascular network seeded with human umbilical vein endothelial cells. Developing EHTs with patent microvascular networks and ample CM-CM connections for proper electrophysiological function has proven to be difficult. Low density collagen scaffolds allow for CMs to electrically couple but do not provide enough structural support to the microvascular network, which become occluded by CM contractions. In contrast, high density collagen scaffolds provide structural support for the vasculature but the CMs are unable to properly electrically couple. In this study, the addition of the fibroblasts increased cellular alignment, provided the microvascular network with structural support for patency up to 2 weeks, and allowed CM electrical coupling within a high density collagen matrix (Roberts et al., 2016). These results illustrate the significance of naturally engineering EHTs with non-cardiomyocytes that provide synergistic benefits to the functional maturity.

## Conclusion

As demonstrated throughout this review, by resembling the developmental cues that occur *in vivo*, natural engineering approaches have led to some major breakthroughs in the *in vitro* maturation of PSC-CMs. The four most prominent physiological factors that synergistically modulate CM behavior are mechanical stimuli, electrical stimuli, extracellular matrix interactions, and non-cardiomyocyte interactions. Studies that more accurately recapitulated the natural developmental program led to the greatest improvements in overall CM maturity.

However, as discussed, even with these current natural engineering approaches, the majority of PSC-CMs within tissue constructs still ultimately remain within the fetal stages of CM development and have yet to reach the maturity of all aspects of late fetal CMs. Therefore, through more studies focusing on *in vivo* fetal CM development, researchers can continue to elucidate how the complex physiological cues interact with CMs to synergistically modulate maturation. This information can then be utilized to establish natural engineering approaches that more closely emphasize the physiological cues throughout the fetal CM development stages and thus lead to greater progress in PSC-CM maturity.

Overall, the timing and practicality of recapitulating the natural developmental program are caveats that need to be considered. *in vivo* human development occurs over a 9 month gestation period, which is drastically longer than the *in vitro* natural engineering approaches that have been implemented. Waiting for the natural developmental time course to take place *in vitro* may not be feasible when producing EHTs for cardiac regenerative purposes. Combining all aspects of the natural developmental program into the engineering of cardiac tissue would be an expensive, time-consuming, and daunting process. PSC-CMs, however, may not need to be matured completely to an adult phenotype before implantation *in vivo*. A “minimally accepted” mature PSC-CM phenotype may be all that is needed for complete maturation to then occur within the *in vivo* environment. Nonetheless, harnessing the significant aspects of the natural developmental paradigm, through natural engineering approaches, will provide researchers with ample opportunities to progress PSC-CM maturation toward a more clinically relevant model for cardiac regeneration.

## Author contributions

GS and JB meet the required authorship criteria for this review article.

## Funding

This work was supported by funding from the National Institutes of Health (HL110328 and HL128745).

### Conflict of interest statement

The authors declare that the research was conducted in the absence of any commercial or financial relationships that could be construed as a potential conflict of interest.
